# Effects of Different Exercise Intensities on Internet Addiction in Adolescents and Young Adults: A Systematic Review and Network Meta‐Analysis

**DOI:** 10.1111/adb.70172

**Published:** 2026-07-06

**Authors:** Min Yu, Yuanyuan Chang, Haobo Kang, Wuyang Mao

**Affiliations:** ^1^ School of Physical Education Hunan University of Arts and Science Changde China; ^2^ School of Physical Education and Health Engineering Taiyuan University of Technology Taiyuan China; ^3^ School of Physical Education Hunan University Changsha China

**Keywords:** anxiety, depression, exercise intensity, internet addiction, negative moods

## Abstract

This study employed a network meta‐analysis to evaluate and compare the efficacy of different exercise intensities (light, moderate and vigorous) on internet addiction and related psychological symptoms in adolescents and young adults. We systematically searched eight databases for relevant randomised controlled trials. A total of 22 studies with 1438 samples were included. A pairwise meta‐analysis indicated that exercise interventions across all intensities were associated with improvements in internet addiction, anxiety and negative moods, with moderate‐intensity additionally improving depression. The results of the network meta‐analysis suggested that light‐intensity exercise (SMD = −2.61, 95% CI [−4.29, −0.93], *p* = 0.002), moderate‐intensity exercise (SMD = −2.74, 95% CI [−4.25, −1.22], *p* < 0.0001) and high‐intensity exercise (SMD = −2.55, 95% CI [−4.88, −0.22], *p* = 0.032) may be associated with a reduction in adolescents' internet addiction. Only moderate‐intensity exercise may be associated with a reduction in depression (SMD = −1.19, 95% CI [−2.20, −0.18], *p* = 0.021) and anxiety (SMD = −1.90, 95% CI [−3.50, −0.30], *p* = 0.02); light‐intensity and high‐intensity exercise were not significantly associated with a reduction in depression and anxiety. Light‐intensity exercise (SMD = −1.63, 95% CI [−2.85, −0.41], *p* = 0.009) and moderate‐intensity exercise (SMD = −1.55, 95% CI [−2.56, −0.54], *p* = 0.003) may be associated with reductions in other negative moods. According to the probability ranking of the likelihood of the optimal intervention effect, moderate‐intensity exercise (SUCRA = 71) ranked highest for interventions in adolescents' internet addiction, depression (SUCRA = 70.2), anxiety (SUCRA = 68.4) and light exercise (SUCRA = 67.9) ranked highest for negative moods. However, owing to the limitations of sample size and the quality of individual studies, the strength of evidence needs to be further validated by more standardised, high‐quality studies.

## Graphical abstract

This study examines how young adults with experience of out‐of‐home care in Finland describe their relationships with caregivers through the lens of care ethics. The findings identify five elements of ethical care: relational continuity, consistency, attentiveness, responsibility and relationality. Based on these findings, the study develops a model of ethical care in out‐of‐home care. The model offers practical guidance for developing relationally grounded practices that strengthen the ethical quality of care work in out‐of‐home care.

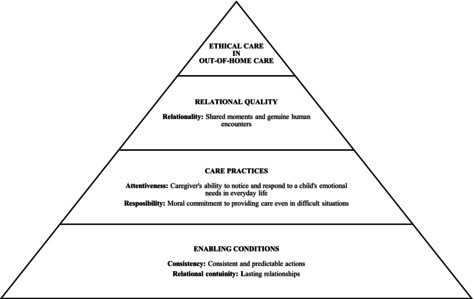

## 1 Introduction

In recent years, with information technology and artificial intelligence sweeping the globe, internet use has penetrated every corner of people's lives, accelerating the reshaping of human lifestyles. According to the International Telecommunication Union (ITU), there will be approximately 5.5 billion internet users in 2024 [1], and digital existence has become an irreversible civilisation process. However, the problem of internet addiction triggered by overimmersion in the virtual space of the internet is gradually emerging. Internet addiction, also known as problematic internet use (PIU), is recognised as a behavioural addiction characterised by chronic inappropriate internet use and has been incorporated into mainstream diagnostic frameworks such as the Diagnostic and Statistical Manual of Mental Disorders (DSM‐5).

International Classification of Diseases (ICD‐11) [2, 3]. Adolescence is a special developmental period, and the tremendous physical, psychological and social changes that occur make adolescents vulnerable to internet addiction [4]. Research has indicated that internet addiction is more prevalent among adolescents [5]. This addiction compromises children's physical and mental health by reducing sleep and physical activity time [6]. Adolescents suffering from internet addiction are prone to physical weakness, dysfunctionality, negative psychiatric problems, weakened immunity and adverse emotional symptoms [7, 8], which seriously jeopardise the normal life of the adolescent population.

Exercise has emerged as a promising, low‐cost intervention for internet addiction, with studies indicating a negative correlation between physical activity and addictive behaviours [9, 10, 11, 12]. Exercise can mitigate internet addiction by regulating neurobiological pathways in the central nervous system and autonomic nervous system, as well as through physical mechanisms that enhance cardiopulmonary function and promote blood circulation [13]. Additionally, exercise may reduce the levels of homovanillic acid and l‐tryptophan, thereby ameliorating the dopamine system disrupted by internet addiction, preventing dysregulation of the brain's reward pathway and alleviating associated side effects [14]. Moreover, exercise can effectively improve the cognitive processing function of internet‐addicted adolescents [15], improve self‐efficacy and self‐control [16], regulate feelings of loneliness, low mood and behavioural problems, reduce anxiety, boost self‐esteem and improve inhibitory control [17, 18, 19, 20], thereby reducing psychological cravings for the internet.

Current academic meta‐analyses on the effects of exercise on adolescent internet addiction interventions are limited and controversial. For example, in a meta‐analysis comparing the intervention effects of exercise and other intervention modalities on adolescent internet addiction, Jiang et al. [21] reported that exercise improved adolescent internet addiction, as did psychotherapy and educational interventions, but did not provide an exact ranking. Some network meta‐analyses have shown that although exercise can improve adolescent internet addiction, it is not the best single intervention [22, 23, 24]. In contrast, some studies have shown that exercise is the best single modality [9, 25, 26]. In a meta‐analysis discussing the intervention effect of exercise on adolescents' internet addiction, some studies categorised exercise into open‐ended and closed‐ended exercises to explore the effects of exercise on different indicators of internet addiction and different degrees of addiction [27]. Zhang et al. [28] explored the intervention effect of different types of exercise on different indicators, and Yan et al. [29] analysed the improvement effect of exercise in terms of different indicators of internet addiction. Specifically, exercises such as dance, yoga, tai chi, basketball, badminton, running and cycling can effectively alleviate internet addiction [27, 30]. Overall, current meta‐analyses on exercise and adolescent internet addiction focus primarily on comparing the effectiveness of exercise versus other interventions for treating adolescent internet addiction and evaluating the efficacy of specific exercises and different exercise types in addressing this issue. Few studies have examined the intrinsic relationship between varying exercise intensities and adolescent internet addiction, along with its associated secondary indicators.

Building upon previous research, the primary objective of this network meta‐analysis is to systematically compare the relative effectiveness of light‐, moderate‐ and vigorous‐intensity exercise interventions, as classified by the American College of Sports Medicine (ACSM) and the Youth Compendium of Physical Activities, in ameliorating internet addiction among adolescents and young adults. Secondary objectives include evaluating the effects of these exercise intensities on frequently co‐occurring psychological symptoms, namely, depression, anxiety and other negative moods. Utilising both pairwise and network meta‐analytic techniques, this study aims to integrate direct and indirect evidence to rank the intervention efficacy of different exercise intensities and combined interventions, thereby identifying potentially optimal exercise prescriptions for addressing adolescent internet addiction and its associated psychological comorbidities.

## 2 Methods

This study was reported in strict accordance with the Preferred Reporting Items for Systematic Reviews and Meta‐Analyses (PRISMA) network meta‐analysis (NMA) guidelines [31]. The review protocol was registered with the International Prospective Register of Systematic Review (PROSPERO; CRD420251005725).

### 2.1 Search Strategy

A computerised search was conducted for randomised controlled trials (RCTs) of exercise prescription interventions for internet addiction in adolescents in the CNKI, Wanfang, VIP, EMBASE, PubMed, Web of Science, EBSCO and Cochrane Library databases. The PubMed/Cochrane and EMBASE databases were searched via ‘Mesh’ and ‘Emtree’ subject terms, respectively, and the search terms included ‘internet addiction’ OR ‘internet addiction disorder’ OR ‘online addiction’ OR ‘social media addiction’ OR ‘addiction, smartphone’ OR ‘internet gaming disorder’ OR ‘pathological internet’ OR ‘computer game addiction’ AND ‘exercise’ OR ‘activity, physical’ OR ‘exercise, acute’ OR ‘exercise, isometric’ OR ‘aerobic exercise’ OR ‘exercise training’ AND ‘randomized controlled trial’ OR ‘random’ OR ‘placebo’ AND ‘adolescent’ OR ‘adolescents’ OR ‘adolescence’ OR ‘teens’ OR ‘teenagers’. Subject terms with free words were used, the search languages were Chinese and English, and the search period was from the construction of each database to July 1, 2025. To maximise the inclusion of relevant studies, the snowball method was used to trace the references of the included literature and related reviews to avoid overlooking relevant studies that might have been lost in the electronic search.

### 2.2 Inclusion Criteria

On the basis of the five criteria of P (participants), I (intervention), C (comparison), O (outcome) and S (study design) contained in a single research institute, this study formulated the criteria for literature retrieval, inclusion, screening and exclusion [32]. The specific criteria were as follows: (1) the subjects were a group of adolescents or young adults (e.g., secondary school and university/college students) diagnosed with internet addiction via a standardised scale or clinical assessment; (2) the experimental interventions included structured exercise programs of any type with a defined intensity. The intensity had to be classified as light, moderate or vigorous according to the ACSM criteria, either directly reported by the study authors or classifiable by our review team based on the provided descriptions (e.g., heart rate [HR], maximal oxygen uptake [VO_2_ max], metabolic equivalent of exercise [MET], or rating of perceived exercise [RPE] intensity); (3) eligible control conditions included no intervention, wait‐list control, treatment as usual or other nonexercise interventions. Studies comparing different exercise intensities were also eligible; (4) the primary outcome was the severity of internet addiction, measured by any validated scale. Secondary outcomes included symptoms of depression, anxiety and other negative moods (e.g., loneliness, stress), also measured by standardised instruments; (5) the study design was a randomised controlled experiment; (6) to ensure the quality and authority of the original research, the Chinese studies to be included should have been published in the database of the Chinese core journals.

### 2.3 Exclusionary Criteria

The exclusion criteria for original studies were (1) duplicates of published literature, theoretical and review studies, dissertations, letters to the editor and abstracts published in conference proceedings; (2) subjects were not adolescents or were not specifically diagnosed with internet addiction; (3) the full text was unavailable after attempting to contact the corresponding authors; (4) the study design was nonrandomised and controlled; (5) the severity of IA was incomplete or unreported; (6) the study was published in a language other than English and Chinese; (7) the intervention did not involve a structured exercise program or the intensity could not be determined and (8) the study was a single‐arm before‐and‐after comparison study.

### 2.4 Classification of Exercise Intensity

The present study classified the exercise intensities included in the RCTs into four categories according to the recommendations of the ACSM and the ‘Youth Compendium of Physical Activities’ [33, 34]:

1. Light
2. Moderate
3. Vigorous
4. No exercise or slight stretching

The metrics used to assess exercise intensity are HR, HR reserve (HRR), VO_2_ max and the RPE [33, 34, 35]. In this study, exercise intensity was assessed by first classifying the exercise intensity against a classification table of exercise intensity on the basis of physiological indices given in the original literature and taking into account the age and physical characteristics of the subjects (e.g., presence of underlying diseases) [33]. If there were no indicators for assessing exercise intensity in the literature, the MET was calculated by comparing the type of exercise described in the literature with the ‘2024 Physical Activity Compendium for Adults’ [36] and supplemented it with the ‘Youth Compendium of Physical Activities’ for younger participants, and the energy expenditure per minute (MET) of exercise was calculated. Exercise intensity was then categorised according to the Exercise Intensity Scale, taking into account the age of the participants [33].

### 2.5 Data Extraction

Literature screening and data extraction were performed independently by two researchers. Two investigators independently extracted all data, with any disagreements resolved by consensus or by consultation with a third author if necessary. The basic information extracted included the name of the first author, year of publication, subject information (sample source, age and sample size of the experimental and control groups), intervention information (type of exercise, intensity, frequency, duration and length of intervention), outcome indicators and evaluation tools. In cases of insufficient information, the authors of the included studies were contacted by e‐mail as needed to obtain missing data.

### 2.6 Outcomes

The primary outcome was the severity of internet addiction, measured by any validated scale. Secondary outcomes included symptoms of depression, anxiety and other negative moods (e.g., loneliness, stress), also measured by standardised instruments.

In this study, due to the heterogeneity of negative emotional indicators associated with internet addiction (except for depression and anxiety) and the lack of sufficient support in the literature, these indicators were combined into a comprehensive measure based on a transdiagnostic perspective. Internet addiction among adolescents and young adults is often comorbid with a range of transdiagnostic emotional distress, which are characterised by conceptual overlap and high comorbidity [37]. Furthermore, integrating various negative emotions aligns with the growing recognition of exercise as a transdiagnostic intervention capable of simultaneously improving mental health across multiple symptom domains [38]. Therefore, regarding the indicator of negative emotions, this study examines the overall effect of exercise on this construct rather than on any single symptom.

### 2.7 Risk of Bias and GRAD Assessment

The quality of the included studies was assessed by two researchers in an independent, double‐blind manner according to the Cochrane Risk of Bias Tool [39]. Given that blinding participants to an exercise intervention is difficult, ‘blinding of participants and personnel’ was not included in the overall risk of bias score. The evaluation included six levels: (1) random allocation method; (2) scheme hiding; (3) blinding method for the statistical analysis of the data; (4) integrity of the resulting data; (5) selective reporting of the research results; and (6) other sources of bias. At each level, high risk of bias, unclear risk of bias and low risk of bias were assessed. In addition, we summarised and evaluated the overall ROB of each study as follows: studies were classified as having low ROB if none of the domains above was rated as high ROB and three or less were rated as unclear risk, and as moderate ROB if one was rated as high ROB or none was rated as high ROB, but four or more were rated as unclear risk, and all other cases were assumed to pertain to high ROB [40]. Any disagreements arising from the evaluation results were resolved after discussion with a third researcher.

We assessed the certainty of evidence for the network estimates of the outcomes with the Grading of Recommendations Assessment, Development and Evaluation (GRADE) framework for network meta‐analysis [41]. The certainty of evidence (high, moderate, low, or very low) was evaluated for each pairwise comparison within the network for the four outcome indicators (internet addiction, depression, anxiety, negative moods).

### 2.8 Data Analysis

This study employed two complementary meta‐analytical approaches to address its research objectives. First, a pairwise meta‐analysis was conducted to synthesise the direct evidence and determine the absolute efficacy of each exercise intensity compared to the no‐intervention group for each outcome. Subsequently, an NMA was performed to integrate both direct and indirect evidence, allowing for the estimation of the relative effects between all pairs of interventions within the network and to rank their comparative effectiveness.

The before and after changes in the experimental and control groups were summarised to estimate the effects. The standard deviation (SD) of changes was calculated via the formula provided in the Cochrane Handbook for Systematic Reviews of Interventions (version 6.3) [42]: $\begin{array}{c} \begin{array}{c} {\mathrm{SD}}_{\text{change}}=\sqrt{{{\mathrm{SD}}^{2}}_{\text{baseline}}+{{\mathrm{SD}}^{2}}_{\text{final}}-\left(2\times \text{Corr}\times {\mathrm{SD}}_{\text{baseline}}\times {\mathrm{SD}}_{\text{final}}\right)} \end{array} \end{array}$. In this study, a meta‐analysis was conducted via Review Manager 5.3 (the Cochrane Collaboration, Copenhagen, Denmark) [43]. Since the original studies were assessed via different scales for the scores of the indicators related to internet addiction in adolescents and the outcome variables were continuous random variables, the standardised mean difference (SMD) was used for the merging of effect sizes [44], and 95% confidence intervals (CIs) were calculated, with 95% CIs providing a range of estimates indicating that there is a 95% probability that the true effect size falls within the interval, thus assessing the precision and reliability of the estimates [45]. Heterogeneity was judged by *I* ^2^ values and *Q* tests, with *I* ^2^ values greater than 50% or *p* values less than 0.1 indicating greater heterogeneity [39]. When the heterogeneity was significant, the random effects model was used; otherwise, the fixed effects model was used. Subgroup analyses included on sample size, population type, exercise intensity, intervention duration, risk of bias, frequency and exercise duration. Sensitivity analyses were also used to test whether the source of heterogeneity originated from one of the original studies, and publication bias was evaluated by assessing corrected funnel plots and performing a Begg's test in Stata 17.0 (StataCorp LLC, College Station, TX). If excessive publication bias was present, the reliability of the results was tested via the cut‐and‐patch method.

This study used Stata 17.0 to conduct a random effects multivariate network meta‐analysis [46] of pooled estimates and 95% CI within a frequency‐based framework in accordance with the current PRISMA NMA guidelines [31]. Network evidence maps were plotted for the different intervention modalities, which consisted of dots and lines, with the dots representing the intervention modality and the size of the dots representing the number of subjects and the lines representing the intervention modality for direct comparisons and the thickness of the lines representing the number of included studies. The network contribution was plotted, and the contribution of each direct comparison was calculated. Inconsistency factors (IFs) for the 95% CI were calculated to assess the consistency of each closed loop, with consistency indicated by a 95% CI lower bound of 0 [47]. Inconsistency modelling was applied to test inconsistency, and consistency modelling was used when inconsistency was not significant (*p* > 0.05) [48]. Nodal splitting analysis was used to check for local inconsistency, and the results were reliable when *p* > 0.05. The intervention effects of the different interventions were ranked and compared via the cumulative probability ranked‐ordered curve (SUCRA) value, which takes values ranging from 0 to 100, where 100 indicates the best treatment without uncertainty and 0 indicates the worst treatment without uncertainty [49]. Thus, higher SUCRA values indicate a more effective exercise intervention. Moreover, publication bias analysis was performed via a network funnel plot.

## 3 Results

### 3.1 Study Selection

**FIGURE 1:**
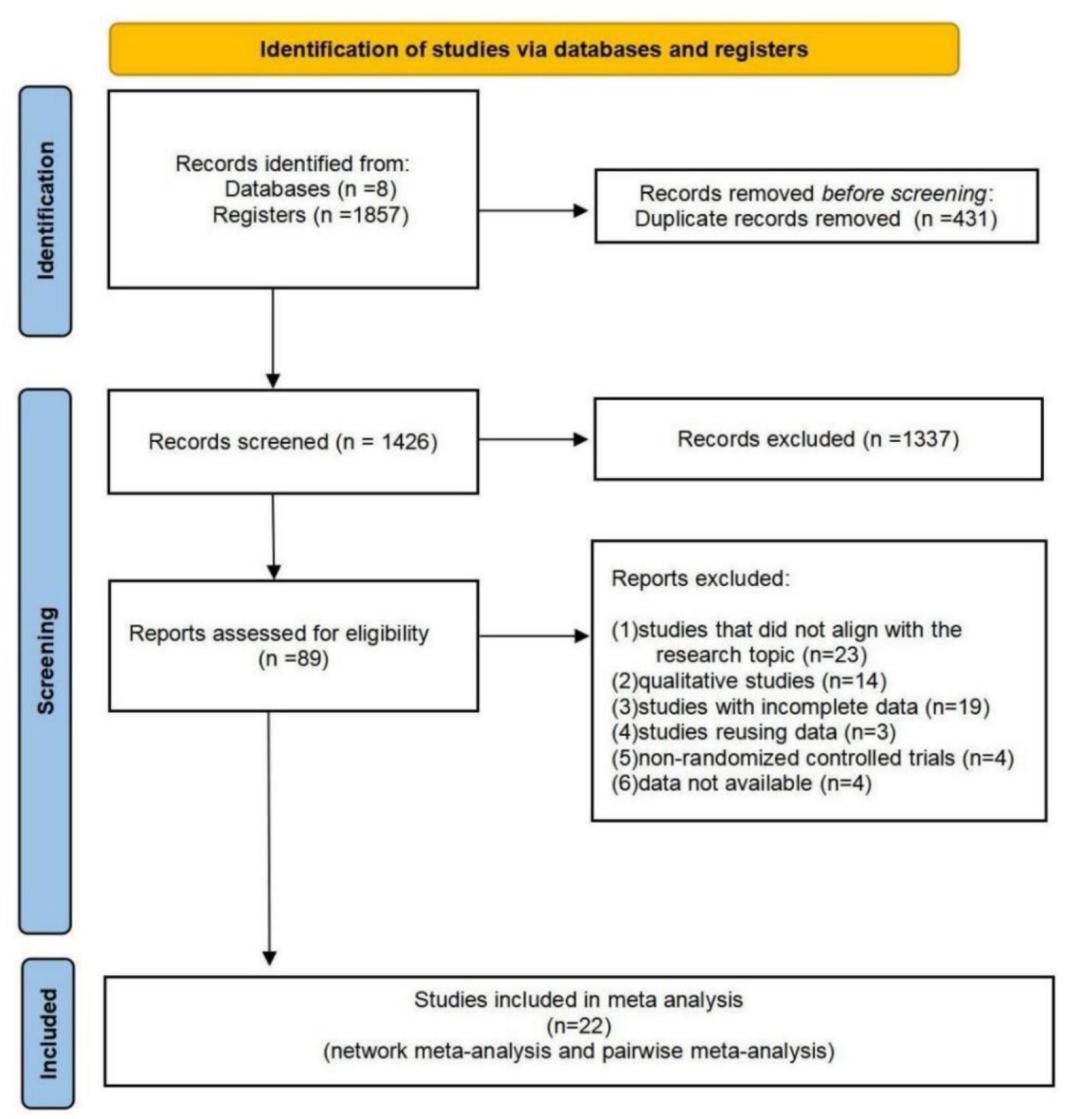
PRISMA flow diagram depicting the study selection process.

The flow diagram reporting trial selection is shown in Figure 1. A total of 1857 potentially eligible articles were identified from eight databases. After 431 duplicates were removed, 1426 articles remained for screening. After screening the titles and abstracts, 1337 articles were removed and 67 articles were removed after the full texts were obtained and read, leaving 22 articles for quantitative synthesis, of which 6 were in English and 16 were in Chinese.

### 3.2 Study Characteristics

The basic information of the included studies is shown in Table 1. These studies were published between 2007 and 2024 and included 16 core papers in Chinese and 6 in English. A total of 1438 adolescents with internet addiction were included in the 22 papers, of which 805 internet‐addicted adolescents were included in the experimental group (light: 241; moderate: 435; vigorous: 129), and 633 were included in the control group. The mean age of the included subjects ranged from 13 to 22 years, encompassing both adolescents and college‐aged young adults. Sixteen studies did not report the ratio of males to females; the majority of the studies recruited participants from universities (*n* = 18), and some studies recruited participants from high schools (*n* = 3) and hospitals (*n* = 1).

**TABLE 1:** Detailed characteristics of the included studies.

Author and published year	Source of participants	Participant characteristics				Means of intervention					Outcome measurements	Outcomes
		Sample size		Age (mean SD)								
		T	C	T	C	T (Intensity)	C	Intervention duration	Frequency (times/week)	Exercise duration (minutes)		
Zhao et al. 2021 [50]	University	49	50	20.14 ± 1.27	19.81 ± 1.21	Aerobic exercise (M) + psychological intervention	Psychological intervention	6 months	2	60	SAS‐C/VAS/HAMD/HAMA	①②③④
		49		20.21 ± 1.19		Aerobic exercise (M)						
Zhang 2009 [51]	University	35	35	21.04	21.04	Aerobic exercise (M) + psychological intervention	—	12 weeks	2	80	Self‐made/STAI	①③④
Yang and Zeng 2017 [52]	University	26	26	19.6 ± 1.2	19.7 ± 1.4	Tai chi (L)	—	16 weeks	4	60	CIAS	①④
Wen and Chen 2020 [53]	University	40	40	20.63 ± 2.06	20.34 ± 1.24	Running (V)	—	8 weeks	3–4	43	CIAS	①④
Liu et al. 2022 [54]	University	31	34	18–22	18–22	Basketball (V)	—	10 weeks	2	60	MPAI	①④
		31		18–22		Qi gong (L)						
Li et al. 2014 [55]	Middle school	27	24	15.41 ± 1.47	15.62 ± 1.78	HIIT (V)	—	10 weeks	3	50–60	IAT	①
Li et al. 2009 [56]	University	16	16	—	—	Badminton (M)	—	8 weeks	3	40–60	IAT/SCL‐90	①②③④
Ge et al. 2015 [57]	University	18	18	21.24 ± 1.08	21.24 ± 1.08	Volleyball (L)	—	18 weeks	3	120	MPAI	①④
Gao et al. 2012 [58]	University	35	34	—	—	Bicycling (M)	—	2 months	5	120	IAT/SCL‐90	①④
Fu and Liu 2016 [59]	University	42	42	20.41 ± 1.37	20.41 ± 1.37	Soccer (M)	—	16 weeks	3	50	IAT	①
Deng 2014 [60]	University	24	24	—	—	Aerobic exercise (M)	—	10 weeks	3	50	CIAS/SCL‐90	①②③④
Xiao et al. 2021 [61]	University	31	34	18.95 ± 0.89	19.71 ± 1.77	Basketball (V)	—	12 weeks	3	90	MPAI/SAS /UCLA‐LS	①③④
		31		19.21 ± 1.02		Baduanjin (L)						
Lu et al. 2020 [62]	University	31	34	19.21 ± 1.02	19.71 ± 1.77	Qi gong (L)	—	12 weeks	2	90	MPAI/SAS/ULS‐8	①③④
Hong et al. 2020 [63]	Hospital	25	25	13–18	13–18	Aerobic exercise (L) + CBT	CBT	14 weeks	1	90	YIAS/BDI/BAI /K‐ARS	①②③④
Lee and Huh 2024 [64]	University	10	10	—	—	AR + aerobic exercise (M)	Aerobic exercise (M)	—	—	—	K‐POMS‐B	②④
Zhang et al. 2024 [65]	University	30	30	20.03 ± 0.56	20.20 ± 0.61	Aerobic exercise (M)	—	8 weeks	3	60	SAS‐SV/SDS /SAS/RSES	①②③④
		30		20.10 ± 0.76		Tai chi (L)						
Zhang et al. 2023 [66]	University	31	31	20.03 ± 0.55	20.23 ± 0.62	Aerobic exercise (M)	—	8 weeks	3	60	IAT/SDS/SAS/FS‐14	①②③④
		31		20.10 ± 0.75		Tai chi (L)						
Zhang et al. 2022 [67]	University	17	20	20.27 ± 1.95	20.27 ± 1.95	Aerobic exercise (M)	—	8 weeks	2	60	MPATS/SAS/SDS	①②③④
Qiu and Zhai 2011 [68]	University	18	18	—	—	E‐sports (L)	—	3 months	7	100–120	IAT	①
Guo et al. 2007 [69]	Middle school	9	9	13–18	13–18	Aerobic exercise (M)	—	8 weeks	1	60	SCL‐90	②③④
		9		13–18		Aerobic exercise (M) + psychological intervention						
Hu and Zhang 2016 [70]	Middle school	49	49	—	—	Aerobic exercise (M)	—	12 months	3	90	CIAS/MMHI	①②③④
Bu 2014 [71]	University	30	30	18–21	18–21	Aerobic exercise (M)	—	6 months	3–5	60–90	MPATS	①④

①, Internet addiction; ②, depression; ③, anxiety; ④, negative moods.

Abbreviations: AR, augmented reality; CBT, cognitive behavioural therapy; L, light‐intensity exercise; M, moderate‐intensity exercise; V, vigorous‐intensity exercise.

The exercise intensities reported in the literature in this study included light (9 groups), moderate (15 groups) and vigorous (4 groups). The exercise types included aerobic exercise, running, ball sports (basketball, volleyball, soccer, badminton), traditional Chinese kung fu (Tai Chi, Qigong, Baduanjin) and e‐sports. Eighteen studies did not use any intervention in the control group. The intervention period ranged from 8 weeks to 12 months, the frequency of interventions ranged from one to seven times per week, and the duration of a single intervention ranged from 43 to 120 min (one study did not report the intervention period, duration or frequency).

The included studies assessed relevant outcomes using various validated scales. A complete list of these measurement tools and their references is provided in the Supporting Information.

### 3.3 Methodological Quality Assessment

**FIGURE 2:**
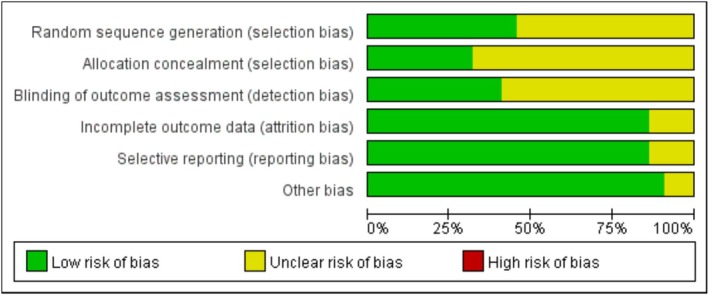
Risk of bias graph.

The methodological quality of the 22 included papers was assessed. According to the risk of bias assessment summary, 86.4% (*n* = 19) of the included studies were low risk, 9.1% (*n* = 2) were medium risk and 4.5% (*n* = 1) were high risk. Details of the risk of bias assessment for each study are shown in Appendix 1, and the risk of bias assessment is summarised in Figure 2.

### 3.4 Pairwise Meta‐Analysis

A pairwise meta‐analysis was first conducted to compare the intervention effects of different exercise intensities on internet addiction, depression, anxiety and other adverse moods compared with a blank control group, and the forest plot is detailed in Appendix 2. Compared with the control, light exercise significantly improved internet addiction (SMD = −1.46, *p* < 0.00001, 95% CI [−2.78, −0.14], *I* ^2^ = 95%), anxiety (SMD = −2.13, *p* = 0.03, 95% CI [−3.11, −1.15], *I* ^2^ = 95%) and other negative moods (SMD = −1.43, *p* = 0.005, 95% CI [−2.43, −0.42], *I* ^2^ = 95%). Moderate‐intensity exercise significantly improved internet addiction (SMD = −2.80, *p* < 0.00001, 95% CI [−3.00001, −1.72], *I* ^2^ = 96%), depression (SMD = −1.52, *p* = 0.007, 95% CI [−2.62, −0.42], *I* ^2^ = 95%), anxiety (SMD = −2.28, *p* = 0.002, 95% CI [−3.73, −0.84], *I* ^2^ = 96%) and other negative emotions (SMD = −1.67, *p* = 0.0006, 95% CI [−2.63, −0.71], *I* ^2^ = 95%). High‐intensity exercise significantly improved internet addiction (SMD = −2.22, *p* = 0.0001, 95% CI [−3.11, −1.15], *I* ^2^ = 95%), anxiety (SMD = −1.89, *p* < 0.00001, 95% CI [−2.48, −1.30]) and other negative emotions (SMD = −1.30, *p* = 0.0002, 95% CI [−1.99, −0.62], *I* ^2^ = 80%). Only light exercise did not significantly ameliorate depression (SMD = −0.34, *p* = 0.06, 95% CI [−0.70, 0.02], *I* ^2^ = 0%). In addition, heterogeneity was not reported because only one study for high‐intensity exercise was included in the anxiety indicator. Sensitivity analyses revealed no significant change in heterogeneity across groups after studies were excluded one by one. A combination of funnel plots (Appendix 3) and Begg's tests revealed publication bias for only the effects of moderate‐intensity exercise on anxiety and internet addiction (*p* < 0.05), and further analyses using cut‐and‐patch methods revealed no significant changes in the results, suggesting that the results were relatively stable.

### 3.5 Results of Subgroup Analysis

We performed subgroup analyses based on sample size, population type, exercise intensity, intervention duration, risk of bias, frequency and exercise duration. Since there were no differences in the quality assessments of the included studies on depression, anxiety and negative emotions, no subgroup analysis was conducted to assess the risk of bias. The results of these analyses are presented in Appendix 3.

The results showed that differences in exercise duration led to different outcomes across all indicators (*p* < 0.05), while differences in intervention duration led to different outcomes in internet addiction (*p* = 0.05), anxiety (*p* = 0.02) and negative emotions (*p* = 0.04). For depression, differences in exercise intensity led to different outcomes (*p* = 0.05). No significant differences were observed in population type, sample size or frequency across all outcomes (*p* > 0.05). However, given the limited number of studies in each subgroup, the results should be interpreted with caution.

### 3.6 Network Meta‐Analysis

To test for differences in effects between exercise intensities, we further conducted a network meta‐analysis.

#### 3.6.1 Network Geometry

**FIGURE 3:**
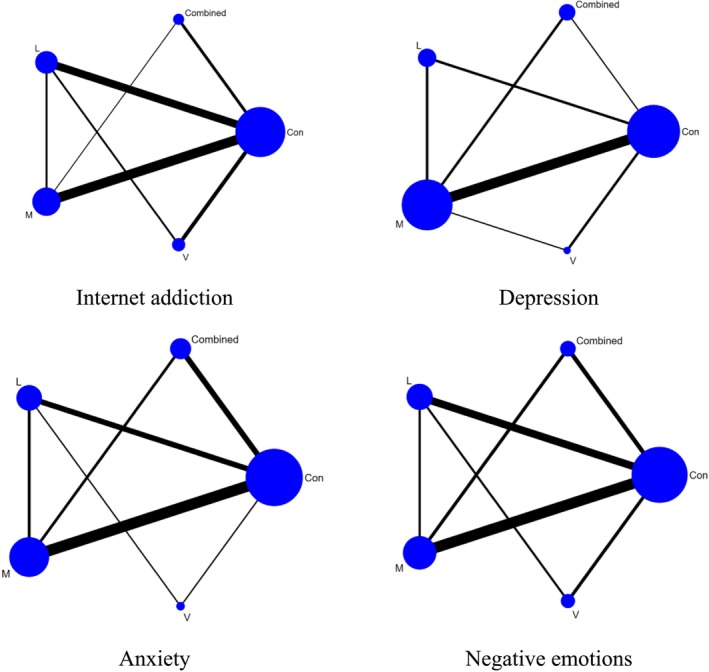
Network geometry of outcomes Abbreviations: L, light‐intensity exercise; M, moderate‐intensity exercise; V, vigorous‐intensity exercise; Con, control group.

As shown in Figure 3, the dots in the figure indicate the number of subjects in each group; larger dots indicate a larger sample size, the lines connecting the dots indicate the number of original studies for each two‐by‐two direct comparison, and thicker lines indicate a larger number of original studies.

#### 3.6.2 Contribution Plot

The contributions of the two‐by‐two direct comparisons of each intervention to the results of the reticulated meta‐analysis and the number of documents for each two‐by‐two direct comparison of interventions are shown in Appendix 5.

#### 3.6.3 Consistency of the Network

Inconsistency between direct and indirect comparisons was assessed via the ring inconsistency test, overall inconsistency test and node‐cutting method, the results of which are presented in Appendix 6, and no significant inconsistency was found for all the results, suggesting that the overall consistency of the included literature is good and that the results are credible.

#### 3.6.4 Results of the Network Meta‐Analysis

Network forest plots showing direct comparisons between interventions are shown in Appendix 7. Summary estimates of the NMA for each outcome indicator of adolescent internet addiction are shown in Table 2. Compared with the control, light exercise (SMD = −2.61, 95% CI [−4.29, −0.93], *p* = 0.002), moderate‐intensity exercise (SMD = −2.74, 95% CI [−4.25, −1.22], *p* < 0.0001) and high‐intensity exercise (SMD = −2.55, 95% CI [−4.88, −0.22], *p* = 0.032) had a significant ameliorative effect on adolescent internet addiction. Moderate‐intensity exercise (SMD = −1.19, 95% CI [−2.20, −0.18], *p* = 0.021) significantly improved depression, whereas light exercise (SMD = −0.77, 95% CI [−2.62, 1.08], *p* = 0.415) and high‐intensity exercise (SMD = −1.19, 95% CI [−2.87, 0.48], *p* = 0.163) were not associated with a statistically significant reduction in depression. Moderate‐intensity exercise (SMD = −1.90, 95% CI [−3.50, −0.30], *p* = 0.02) had a significant ameliorating effect on anxiety, while there was no significant effect between light (SMD = −1.79, 95% CI [−4.00, 0.41], *p* = 0.111) and high‐intensity exercise (SMD = −2.31, 95% CI [−6.49, 1.86], *p* = 0.277). Light exercise (SMD = −1.63, 95% CI [−2.85, 0.41], *p* = 0.009) and moderate‐intensity exercise (SMD = −1.55, 95% CI [−2.56, 0.54], *p* = 0.003) had significant ameliorative effects on other negative moods, whereas high‐intensity exercise (SMD = −1.62, 95% CI [−3.42, 0.18], *p* = 0.078) had no significant effect. There was no significant effect between the combined intervention on internet addiction (SMD = −1.85, 95% CI [−4.56, 0.86], *p* = 0.181), depression (SMD = −0.63, 95% CI [−3.28, 2.02], *p* = 0.643), anxiety (SMD = −0.96, 95% CI [−3.17, 1.24], *p* = 0.392), or negative mood (SMD = −1.25, 95% CI [−2.72, 0.23], *p* = 0.097) in adolescents. In addition, no significant differences were found between light, moderate and vigorous exercise across all outcomes.

**TABLE 2:** Network meta‐analysis matrix of internet addiction, depression, anxiety and negative emotions.

Internet addiction				
Combined				
0.76 (−2.41, 3.93)	L			
0.88 (−2.07, 3.84)	0.13 (−1.98, 2.23)	M		
0.70 (−2.87, 4.27)	−0.06 (−2.64, 2.52)	−0.19 (−2.93, 2.56)	V	
−1.85 (−4.56, 0.86)	−2.61 (−4.29, −0.93)	−2.74 (−4.25, −1.22)	−2.55 (−4.88, −0.22)	Con

Depression				
Combined				
0.14 (−3.01, 3.29)	L			
0.56 (−2.09, 3.21)	0.42 (−1.43, 2.27)	M		
0.57 (−2.49, 3.62)	0.42 (−1.97, 2.82)	0.00 (−1.68, 1.69)	V	
−0.63 (−3.28, 2.02)	−0.77 (−2.62, 1.08)	**−1.19 (−2.20, −0.18)**	−1.19 (−2.87, 0.48)	Con

Anxiety				
Combined				
0.83 (−2.22, 3.88)	L			
0.94 (−1.50, 3.38)	0.11 (−2.33, 2.54)	M		
1.35 (−3.34, 6.05)	0.52 (−3.65, 4.69)	0.42 (−3.97, 4.80)	V	
−0.96 (−3.17, 1.24)	−1.79 (−4.00, 0.41)	**−1.90 (−3.50, −0.30)**	−2.31 (−6.49, 1.86)	Con

Negative emotions				
Combined				
0.38 (−1.50, 2.26)	L			
0.30 (−1.24, 1.84)	−0.08 (−1.55, 1.39)	M		
0.37 (−1.95, 2.69)	−0.01 (−1.92, 1.90)	0.07 (−1.96, 2.10)	V	
−1.25 (−2.72, 0.23)	**−1.63 (−2.85, −0.41)**	**−1.55 (−2.56, −0.54)**	−1.62 (−3.42, 0.18)	Con

*Note:* Effects are expressed as the effect size (95% CI) between interventions. Values in bold indicate that the longitudinal intervention has a more significant reduction impact than the horizontal intervention.

Abbreviations: L, light‐intensity exercise; M, moderate‐intensity exercise; V, vigorous‐intensity exercise; Con, control group.

Forest plots with 95% CIs and 95% prediction intervals of eligible comparisons of internet addiction, depression, anxiety and negative emotions are shown in Appendix 8.

#### 3.6.5 Intervention Effect Ranking

The SUCRA values for each intervention in the network for the four outcome indicators are shown in Table 3, and the probability ranking graphs are shown in Appendix 9. The SUCRA values are the probabilities that each intervention is at its best in the network, with larger values indicating higher ranking probabilities. According to the results of the SUCRA probability ranking, moderate‐intensity exercise (SUCRA = 71) is most likely to be the best exercise intensity for intervening in adolescents' internet addiction, followed by light exercise (SUCRA = 66.3); the best exercise intensity for intervening in depression is most likely to be moderate‐intensity exercise (SUCRA = 70.2), followed by high‐intensity exercise (SUCRA = 67.4). The optimal exercise intensity for intervention for anxiety was most likely moderate‐intensity exercise (SUCRA = 68.4), followed by high‐intensity exercise (SUCRA = 68.2), but the difference was not significant; for negative emotions, light exercise (SUCRA = 67.9) was most likely to be the optimal intervention intensity, followed by high‐intensity exercise (SUCRA = 65.3).

**TABLE 3:** Ranking of exercise interventions in order of effectiveness.

Internet addiction (20 studies, *N* = 1391)		Depression (10 studies, *N* = 643)		Anxiety (11 studies, *N* = 854)		Negative emotions (19 studies, *N* = 1267)	
Interventions	SUCRA	Interventions	SUCRA	Interventions	SUCRA	Interventions	SUCRA
M	71	M	70.2	M	68.4	L	67.9
L	66.3	V	67.4	V	68.2	V	65.3
V	63.4	L	50.4	L	62.9	M	64.2
Combined	46.4	Combined	46.8	Combined	40.3	Combined	50.4
Con	2.8	Con	15.2	Con	10.1	Con	2.3

#### 3.6.6 Risk of Bias Across Studies

The funnel plots used to examine the outcome indicators for the presence of NMA publication bias are shown in Appendix 10. The funnel plots for internet addiction, anxiety and negative emotions exhibited some asymmetry, which may suggest the presence of publication bias or small‐study effects. In contrast, the funnel plot for depression was roughly symmetrical. The asymmetry observed for the primary outcome and most secondary outcomes indicates that small studies with larger effect sizes may be overrepresented in the literature, which could lead to overestimation of the true intervention effects. This finding warrants cautious interpretation of the network meta‐analysis estimates.

#### 3.6.7 GRADE Assessment

Appendix 11 shows the SUCRA rankings of the GRADE assessments and interventions for each comparison for each of the four indicators. Overall, it was low to very low for most of the comparisons in internet addiction and moderate to low for the comparisons in depression, anxiety and negative emotions. Internet addiction had a low rank rating on the SUCRA rankings and a medium rating on the remaining three indicators.

## 4 Discussion

Twenty‐two randomised controlled studies from eight databases were included in this study. Direct pairwise meta‐analysis was utilised to assess the effects of different exercise intensities on internet addiction, depression, anxiety and related negative emotions in adolescents compared with a blank control group. Network meta‐analysis of the included studies analysed direct and indirect comparisons between the different interventions and ranked their intervention effects on four outcome indicators.

### 4.1 Pairwise Meta‐Analysis

The results indicated that exercise was associated with improvements in adolescents' internet addiction, depression, anxiety and related negative emotions compared with the blank control group. In this study, the indicators of negative emotions (except depression and anxiety) associated with internet addiction were combined into one outcome indicator because of their fragmentation and insufficient support in the literature. These findings are similar to the findings of Yan et al.'s [29] study that exercise is effective in improving internet addiction (SMD = −1.25, 95% CI [−1.51, −0.99]), depression (SMD = −0.56, 95% CI [−0.99, −0.13]), anxiety (SMD = −1.30, 95% CI [−2.03, −0.56]) and other negative emotions in college students. The observed effect size for exercise on internet addiction was also similar to the findings of Wu et al. [25] (SMD = −1.68, 95% CI [−1.90, −1.46]). Subgroup analysis by exercise intensity indicated that light‐intensity exercise was not associated with a statistically significant reduction in depression (SMD = −0.34, *p* = 0.06, 95% CI [−0.70, 0.02]), while the evidence for high‐intensity exercise on depression was insufficient due to the limited number of original studies.

Significant differences were observed in the subgroup analyses of exercise duration and intervention duration (excluding depression). We found that the effect sizes were larger for interventions lasting longer than 8 weeks and exercise sessions lasting longer than 60 min. This may be because long‐term physical exercise can induce lasting neurobiological adaptations (such as increased hippocampal volume, brain‐derived neurotrophic factor expression, and hypothalamus–pituitary–adrenal axis regulation), which improve cognitive function and enhance emotional regulation [72]. At the same time, sustained physical exercise may help establish stable exercise habits, thereby reducing reliance on the internet. Furthermore, longer exercise sessions may provide more opportunities for social interaction, helping to prevent loneliness and social isolation, particularly in group settings [73].

### 4.2 Network Meta‐Analysis

A further meta‐analysis of the included studies suggested that moderate‐intensity exercise was associated with reductions in internet addiction (SMD = −2.74, 95% CI [−4.25, −1.22], *p* < 0.0001), depression (SMD = −1.19, 95% CI [−2.20, −0.18], *p* = 0.021), anxiety (SMD = −1.90, 95% CI [−3.50, −0.30], *p* = 0.02) and negative mood (SMD = −1.55, 95% CI [−2.56, −0.54], *p* = 0.003) in adolescents compared with those in the control group. Light‐intensity exercise was associated with reductions in internet addiction (SMD = −2.61, 95% CI [−4.29, −0.93], *p* = 0.002) and negative mood (SMD = −1.63, 95% CI [−2.85, −0.41], *p* = 0.009), whereas high‐intensity exercise was associated with a reduction only in internet addiction (SMD = −2.55, 95% CI [−4.88, −0.22], *p* = 0.032).

For the indicator of internet addiction, the observation that exercise interventions were associated with improvements aligns with findings from prior meta‐analyses [9, 21, 22, 23, 24, 25, 28, 30]. However, these studies primarily compared exercise to other modalities or focused on exercise types, without delineating the role of intensity. The present study extends this evidence by suggesting that moderate‐intensity exercise may rank highest among the intensities examined. Furthermore, our conclusion that moderate‐intensity exercise appears optimal is consistent with the principles of exercise prescription for mental health proposed by other researchers [45, 74], who suggest that moderate‐intensity activity often offers the best balance between physiological stimulus and psychological enjoyment, leading to higher adherence and greater overall benefit. In contrast, the present findings differ somewhat from those of Zhang et al. [75], whose network meta‐analysis based on the CIAS scale did not find a significant improvement effect of exercise on internet addiction compared to other interventions. This discrepancy may be attributed to key methodological differences. Zhang et al. (2022) pooled all exercise interventions into a single node, which may have obscured intensity‐specific effects. Our finer‐grained analysis suggests that lumping all exercise together may mask the specific efficacy of appropriately dosed interventions. Furthermore, the outcome measures differed; we incorporated multiple validated scales, which may capture a broader construct of internet addiction.

For other outcome indicators, our finding that exercise was associated with improvements in depression and anxiety is strongly supported by a large body of literature. For instance, a comprehensive network meta‐analysis concluded that exercise is an effective treatment for depression [74]. Wang and Li [20] found that moderate‐intensity aerobic exercise produced the most significant improvement in adolescent depression. Similarly, Zhang et al. [28] and Lu et al. [76] reported that exercise significantly improved depression, loneliness and other negative moods induced by internet addiction in adolescents, which aligns with the patterns observed for moderate‐intensity exercise in the present study. However, studies examining the effects of different exercise intensities on mental health indicators such as depression and anxiety have found that all exercise intensities significantly alleviate these conditions [45]. Moreover, the greater the exercise intensity, the more pronounced the improvement [77]. Moderate‐to‐high‐intensity exercise effectively improves negative emotions in obese individuals [78]. Conversely, other research has indicated that moderate‐intensity exercise may have limited effects on depression among college students [79]. This discrepancy with the present findings may stem from methodological differences, variations in study populations and differing research perspectives.

The SUCRA probability rankings suggested that moderate‐intensity exercise ranked highest for internet addiction (SUCRA = 71), depression (SUCRA = 70.2) and anxiety (SUCRA = 68.4), while light exercise ranked highest for other negative moods (SUCRA = 67.9). Moderate‐intensity exercise may act through multiple interrelated pathways: from a neurobiological perspective, its aerobic properties promote prefrontal cortex function and enhance self‐control [80]. Simultaneously, moderate metabolic equivalents maintain dynamic homeostasis of cortisol levels, whereas the stress response resulting from high‐intensity exercise [81, 82] may exacerbate fluctuations in negative mood. In addition to these biological mechanisms, the social context of exercise also plays a significant role. Moderate‐intensity exercise in children and adolescents frequently occurs in group settings, which may enhance participants' self‐efficacy and social support [72], while providing protection against loneliness and social isolation [83]. This social dimension likely complements the neurobiological and endocrine pathways, collectively contributing to the overall therapeutic effects. In contrast, light exercise may stimulate a balanced release of endogenous neurohormones and a range of natural health restoration mechanisms but relies on subjective regulation with the participant's mind and body and thus may have limited interventional effects on the core symptoms of internet addiction [84]. This limitation, however, does not preclude a superior effect on the more transient symptoms captured by our ‘negative moods’ composite, for which light‐intensity exercise ranked highest in our SUCRA analysis. A possible explanation is that clinical depression and anxiety may require the potent physiological stimulus of moderate‐intensity exercise, whereas many light‐intensity interventions in our network, such as tai chi, qigong, and baduanjin, emphasise breath regulation and meditative movement that may uniquely alleviate daily loneliness, stress and tension through enhanced parasympathetic tone [72]. Furthermore, the positive affective experience of lower‐intensity activity may promote higher daily adherence, yielding cumulative mood‐regulating benefits that buffer everyday negative emotions [85]. Given the exploratory nature of the composite outcome, this interpretation warrants further validation.

The funnel plot asymmetry observed for internet addiction, anxiety and negative emotions in the network meta‐analysis prompts consideration of why such bias may be particularly pronounced in this literature. Several interrelated factors likely contribute. First, the predominance of small, single‐centre trials, many of which lack sufficient statistical power, increases the likelihood that only those with large, statistically significant effects are published. Second, selective outcome reporting appears common: trials often measure multiple psychological constructs but preferentially report positive findings, especially for secondary outcomes like anxiety and negative moods. Third, while blinding of participants and personnel is inherently challenging in exercise trials, as acknowledged in our risk of bias assessment, other methodological limitations are prevalent. These include inadequate allocation concealment, lack of blinding of outcome assessors and absence of preregistered protocols. Such limitations can introduce performance and detection bias, potentially inflating the observed effect sizes. These factors collectively suggest that the published evidence base may overestimate the true benefits of exercise, particularly for outcomes other than the primary one. Consequently, although the SUCRA rankings provide a relative ordering of interventions, the absolute rankings should be interpreted with caution. Nonetheless, the consistency of the network model and the absence of significant local inconsistency provide some reassurance.

### 4.3 Strengths and Limitations

This study focuses on analysing the improvement effects of different exercise intensities on adolescents' internet addiction and related indices on the basis of previous studies and ranks their intervention effects with unified scale scores to further compare and verify the intervention effects of different exercise intensities on adolescents' internet addiction. This network meta‐analysis provides a comprehensive synthesis of the available evidence on exercise intensity for adolescent internet addiction. The findings offer preliminary insights into the comparative efficacy of light‐, moderate‐ and vigorous‐intensity exercise interventions across the core and associated symptoms of adolescents' internet addiction. As anticipated, exercise intervention was associated with improvements compared to control conditions. Furthermore, the analysis yielded a hierarchy of estimated effects: moderate‐intensity exercise ranked highest for internet addiction, depression and anxiety in the present analysis, while light‐intensity exercise ranked first for negative moods in the present analysis. These findings are consistent with our prespecified objective of ranking intervention effects and offer preliminary insights that may help inform the question of which exercise intensity may be most suitable for clinicians and policymakers.

This study has several limitations that should be considered when interpreting the findings. First, the methodological quality of some included studies was suboptimal. The majority of trials did not adequately report the methods of random sequence generation and allocation concealment, and blinding of participants and personnel was largely impossible, which may introduce performance and selection bias. This likely leads to an overestimation of the true intervention effects and affects the internal validity of our conclusions. Second, the included studies were published only in English and Chinese, excluding studies published in other languages. This language bias may have omitted relevant data from other regions, and the generalisability of our findings may be limited to cultural contexts similar to those of the included studies. Consequently, the estimated effect sizes and ranking of interventions might not be universally applicable across different ethnic and cultural populations. Third, this study used the scores of the included outcome indicators as evaluation indicators, but there may be comparability issues due to the inconsistency of the evaluation tools across studies, for which SMDs were used to combine the effect sizes to minimise the impact of differences. Fourth, the generalisability of our findings to younger adolescents is limited. Only 3 of the 22 included studies recruited middle school participants; the remainder were conducted with college students. Therefore, caution is warranted when extrapolating to younger populations, and future studies should include more diverse age groups. Fifth, we acknowledge that the ‘negative moods’ composite remains an exploratory construct that may obscure symptom‐specific intervention effects. Future studies should report specific psychological constructs alongside global scales to enable more precise, symptom‐level conclusions. Finally, the asymmetry observed in the funnel plots for internet addiction, anxiety and negative emotions may reflect publication bias or small‐study effects, where smaller studies with larger effect sizes are more likely to be published. Such biases could lead to overestimation of the pooled effect estimates. However, it is important to note that the network consistency tests did not reveal significant inconsistency, and trim‐and‐fill analyses in the pairwise meta‐analysis did not substantially alter the estimates, suggesting that the results retain some degree of robustness. Nevertheless, the possibility of overestimation cannot be entirely ruled out, and the findings should be interpreted with appropriate caution. Notwithstanding these limitations, the consistency of our main findings across different outcomes and the application of advanced statistical methods provide some support for the potential benefits of moderate‐intensity exercise for adolescents' internet addiction.

## 5 Conclusion

In conclusion, this network meta‐analysis suggests that exercise interventions, particularly of moderate intensity, may be beneficial for reducing internet addiction and associated psychological symptoms in adolescents and young adults. However, due to the funnel plot asymmetry observed in the network meta‐analysis, which may indicate publication bias or small‐study effects, these findings should be considered preliminary. The effect sizes reported may be overestimated, and future high‐quality, large‐scale RCTs are needed to confirm the true efficacy.

The results provide a rationale for future high‐quality, large‐scale RCTs to confirm the efficacy of exercise, particularly at moderate intensity, and to establish more credible effect size estimates before definitive clinical recommendations can be made.

## Author Contributions

**Min Yu:** writing – review, writing – original draft, visualisation, data curation. **Yuanyuan Chang:** visualisation, data curation, conceptualisation. **Haobo Kang:** validation, visualisation. **Wuyang Mao:** writing – review, validation, supervision.

## Funding

Hunan Provincial Basic Education Teaching Reform Research Project: “Development and Practice of School‐Based Curriculum for Exercise Intervention in After‐School Services in Primary Schools under the ‘Health First’ Concept” (No. 25JGYB0441).

## Conflicts of Interest

The authors declare no conflicts of interest.

## Supporting information


**Data S1:** Supporting Information.

**Appendix 1** Risk of bias assessment.
Appendix 2. Forest plots for each pairwise comparison of internet addiction.
Appendix 3. Subgroup analysis of internet addiction, depression, anxiety and negative emotions.
Appendix 4. Funnel plot of internet addiction, depression, anxiety and negative emotions in pairwise meta‐analysis.
Appendix 5. Contributions of direct and indirect comparisons to NMA and the number of studies of each direct comparison of internet addiction, depression, anxiety and negative emotions (A, control; B, light; C, moderate; D, vigorous; E, combined).
Appendix 6. Inconsistency of internet addiction, depression, anxiety and negative emotions tested by loop‐specific heterogeneity estimates, inconsistency model and node splitting analysis.
Appendix 7. Network forest.
Appendix 8. Forest plots of eligible comparisons of internet addiction, depression, anxiety and negative emotions.
Appendix 9. Area under the curve for cumulative ranking probability of each intervention on internet addiction, depression, anxiety and negative emotions.
Appendix 10. The funnel plot graphics of internet addiction, depression, anxiety and negative emotions in NMA.
Appendix 11. GRADE assessment.

## Data Availability

Data sharing is not applicable to this article as no datasets were generated or analysed during the current study.
