# Supplementary material for: Effects of Different Exercise Intensities on Internet Addiction in Adolescents and Young Adults: A Systematic Review and Network Meta‐Analysis
Source: Addict Biol. 2026 Jul 6;31(7):e70172. doi: 10.1111/adb.70172 (PMC13337322; doi:10.1111/adb.70172)
Supplement: Supplementary file 1 — Data S1: Supporting Information. [file ADB-31-e70172-s002.docx]

For inclusion in the study, indicators were measured. The scales used to measure internet addiction include the Smartphone Addiction Scale (SAS-C)^1^, the Chinese internet Addiction Scale (CIAS)^2^, the Mobile Phone Addiction Index (MPAI), the Smartphone Addiction Scale-Short Version (SAS-SV)^3^, the internet Addiction Test (IAT)^4^, the Young internet Addiction Scale (YIAS)^5^, and the Mobile Phone Addiction Tendency Scale (MPATS)^6^. One study developed scales based on Widyanto's^7^and Sue-Huei Chen's^8^ maturation scales. The scales used to measure depression include the Hamilton Depression Scale (HAMD)^9^, Symptom Checklist 90 (SCL-90)^10^, Beck Depression Inventory (BDI)^11^, Korean version of the Profile of Mood States (K-POMS-B)^12^, Self-Depression Scale (SDS)^13^, and Mental Healthiness Inventory for Middle School Students (MHHI)^14^. Scales measuring anxiety include the Hamilton Anxiety Scale (HAMA)^15^, the State-Trait Anxiety Inventory (STAI)^16^, the SCL-90, the Self-Assessment Anxiety Scale (SAS)^17^, and the Beck Anxiety Inventory (BAI)^11^. Scales that measure other adverse emotions associated with internet addiction include the Visual Analog Scale (VAS)^18^, STAI, CIAS, MPAI, SCL-90, UCLA Loneliness Scale (UCLA-LS)^19^, ULS-8 Loneliness Scale^20^, Korean ADHD Rating Scale (K-ARS)^21^, K-POMS-B, Rosenberg self-esteem scale (RSES)^22^, Fatigue Scale-14 (FS-14)^23^, MNHI, MPATS.

1. Su S, Pan TT, Liu QX, Chen XW, Wang YJ, Li MY. Development of the Smartphone Addiction Scale for College Students. *Chinese Mental Health Journal*. 2014;28:392–397.
2. Bai Y, Fan FM. A Study on the Internet Dependence of College Students: the Revising and Applying of a Measurement. *Psychological Development and Education*. 2005;4:99–104.
3. Zhang KX, Zhang XQ, Zhu YJ, Wei HY, Wang TL, Lu XY, et al. Comparative study on the reliability, validity and application of three Mobile Phone Addiction Scales based on college students. *Modern Preventive Medicine*. 2021;48:3176–3181.
4. Young K. Caught in the Net: How to Recognize the Signs of internet Addiction--and a Winning Strategy for Recovery. 1998. Available from: http://www.semanticscholar.org/paper/2ffcad5280fba640eda2cb2216650a953ad76a50
5. YOUNG Kimberly S. Internet Addiction: The Emergence of a New Clinical Disorder. *Cyberpsychology & Behavior*. 2009;1:237–244.
6. Xiong J, Zhou ZK, Chen W, You ZQ, Zhai ZY. Development of the Mobile Phone Addiction Tendency Scale for College Students. *Chinese Mental Health Journal*. 2012;26:222–225.
7. Widyanto L, McMurran M. The psychometric properties of the internet addiction test. *Cyberpsychology & Behavior: The Impact of the internet, Multimedia and Virtual Reality on Behavior and Society*. 2004;7:443–450.
8. Chen SH, Weng LJ, Su YJ, Wu HM, Yang PF. Development of a Chinese internet Addiction Scale and Its Psychometric Study. *Chinese Psychological Journal*. 2003;45:279–294.
9. Li WB, Xu MZ, Jia FJ, Gao YL. Clinical application of the 6-item version of the Hamilton Depression Inventory. *Chinese Journal of Nervous and Mental Diseases*. 2007;2:119–120.
10. Derogatis LR, Lipman RS, Covi L. SCL-90: An outpatient psychiatric rating scale--preliminary report. *Psychopharmacology Bulletin*. 1973;9:13–28.
11. Beck AT, Epstein N, Brown G, Steer RA. An inventory for measuring clinical anxiety: Psychometric properties. *Journal of Consulting and Clinical Psychology*. 1988;56:893–897.
12. Yeun EJ, Shin-Park KK. Verification of the profile of mood states-brief: Cross-cultural analysis. *Journal of Clinical Psychology*. 2006;62:1173–1180.
13. Dunstan DA, Scott N, Todd AK. Screening for anxiety and depression: Reassessing the utility of the Zung scales. *BMC Psychiatry*. 2017;17:329.
14. Wang JS, Li Y, He ES. Development of a mental health scale for Chinese secondary school students and its standardization. *Science of Social Psychology*. 1997;4:15–20.
15. Hamilton M. The assessment of anxiety states by rating. *The British Journal of Medical Psychology*. 1959;32:50–55.
16. Spielberger CD. Theory and research on anxiety. Anxiety and behavior. *Academic press*.1966.
17. Zung WW. A rating instrument for anxiety disorders. *Psychosomatics*. 1971;12: 371–379.
18. Zhu TM, Li H, Du YP, Zheng Z, Jin RJ. Intervention on network craving and encephalofluctuogram in patients with internet addiction disorder:a randomized controlled trial. *Chinese Acupuncture & Moxibustion*. 2011;31.
19. Wu CH, Yao G. Psychometric analysis of the short-form UCLA Loneliness Scale (ULS-8) in Taiwanese undergraduate students. P*ersonality & Individual Differences*. 2008;44:1762–1771.
20. Hays R, Dimatteo MR. A Short-Form Measure of Loneliness. *Journal of Personality Assessment*. 1987;51:69–81.
21. DuPaul George J. Parent and Teacher Ratings of ADHD Symptoms: Psychometric Properties in a Community-Based Sample. *Journal of Clinical Child & Adolescent Psychology*.1991;20:245–253.
22. Jingyu S, Lu W, Yuhong Y, Na S, Xudong Z, Fazhan C. Family Impacts on Self-Esteem in Chinese College Freshmen. *Frontiers in Psychiatry*. 2017;8:279-.
23. Chalder T, Berelowitz G, Pawlikowska T, Watts L, Wallace EP. Development of a fatigue scale. *Journal of Psychosomatic Research*. 1993;37:147–153.
