# Supplementary material for: Effects of Different Exercise Intensities on Internet Addiction in Adolescents and Young Adults: A Systematic Review and Network Meta‐Analysis
Source: Addict Biol. 2026 Jul 6;31(7):e70172. doi: 10.1111/adb.70172 (PMC13337322; doi:10.1111/adb.70172)
Supplement: Supplementary file 2 — Appendix 1 Risk of bias assessment. Appendix 2. Forest plots for each pairwise comparison of internet addiction. Appendix 3. Subgroup analysis of internet addiction, depression, anxiety and negative emotions. Appendix 4. Funnel plot of internet addiction, depression, anxiety and negative emotions in pairwise meta‐analysis. Appendix 5. Contributions of direct and indirect comparisons to NMA and the number of studies of each direct comparison of internet addiction, depression, anxiety and negative emotions (A, control; B, light; C, moderate; D, vigorous; E, combined). Appendix 6. Inconsistency of internet addiction, depression, anxiety and negative emotions tested by loop‐specific heterogeneity estimates, inconsistency model and node splitting analysis. Appendix 7. Network forest. Appendix 8. Forest plots of eligible comparisons of internet addiction, depression, anxiety and negative emotions. Appendix 9. Area under the curve for cumulative ranking probability of each intervention on internet addiction, depression, anxiety and negative emotions. Appendix 10. The funnel plot graphics of internet addiction, depression, anxiety and negative emotions in NMA. Appendix 11. GRADE assessment. [file ADB-31-e70172-s001.docx]

## Effects of Different Exercise Intensities on Internet Addiction in Adolescents and Young Adults: a Systematic Review and Network Meta-analysis

**Min Yu1,Yuanyuan Chang2, Haobo Kang2, Wuyang Mao3,***

^1^ School of Physical Education,Hunan University of Arts and Science, Changde, China

^2^ School of Physical Education and Health Engineering, Taiyuan University of Technology, Taiyuan, China

^3^ School of Physical Education, Hunan University, Changsha,China

*Correspondence: Email address: 19376668796@163.com

**Appendix 1. Risk of bias assessment**

**
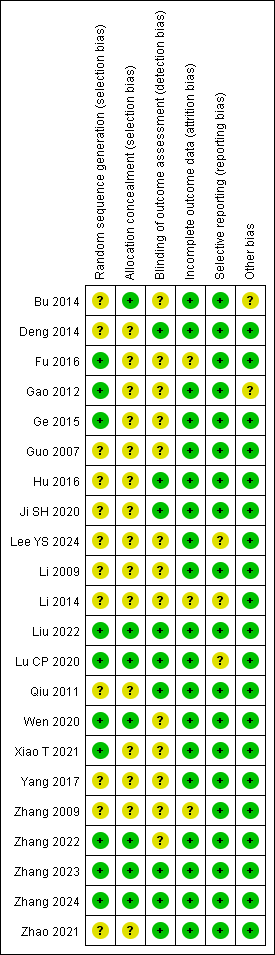
**

Figure 1 Risk of bias summary

**Appendix 2.** Forest plots for each pairwise comparison of Internet addiction


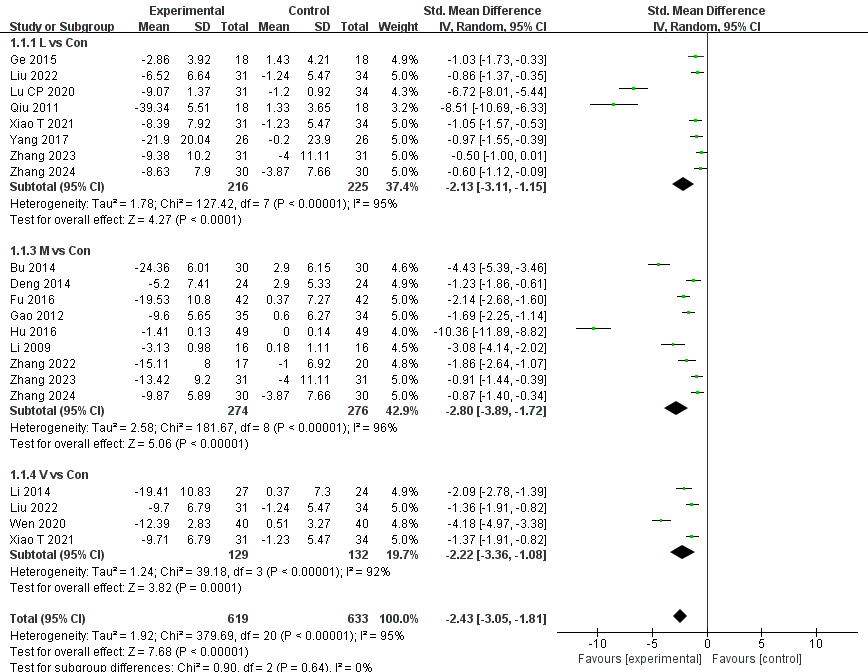


Appendix 2-1 forest plot of Internet addiction


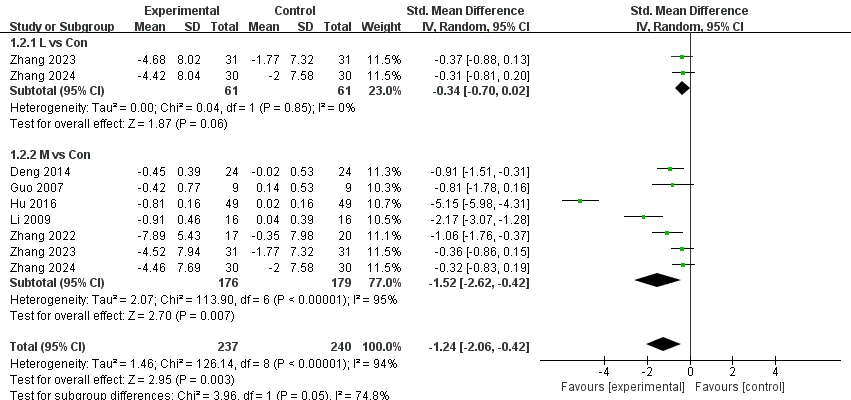


Appendix 2-2 forest plot of depression


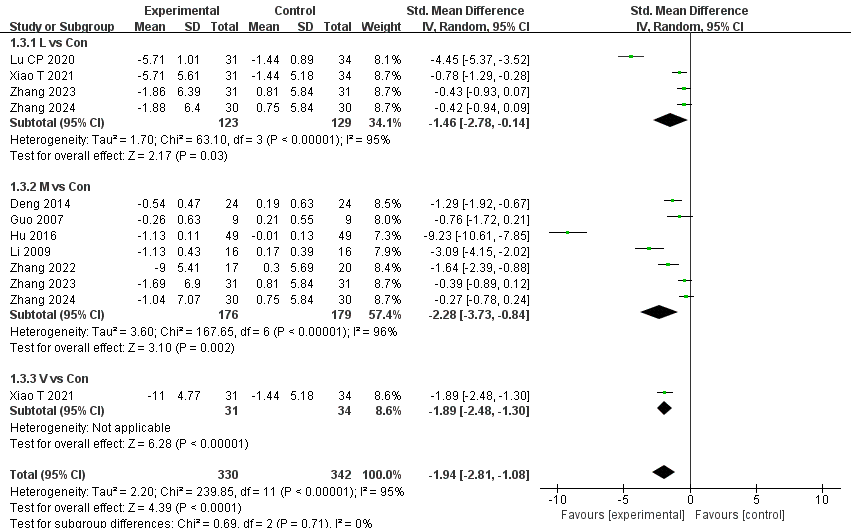


Appendix 2-3 forest plot of Anexity


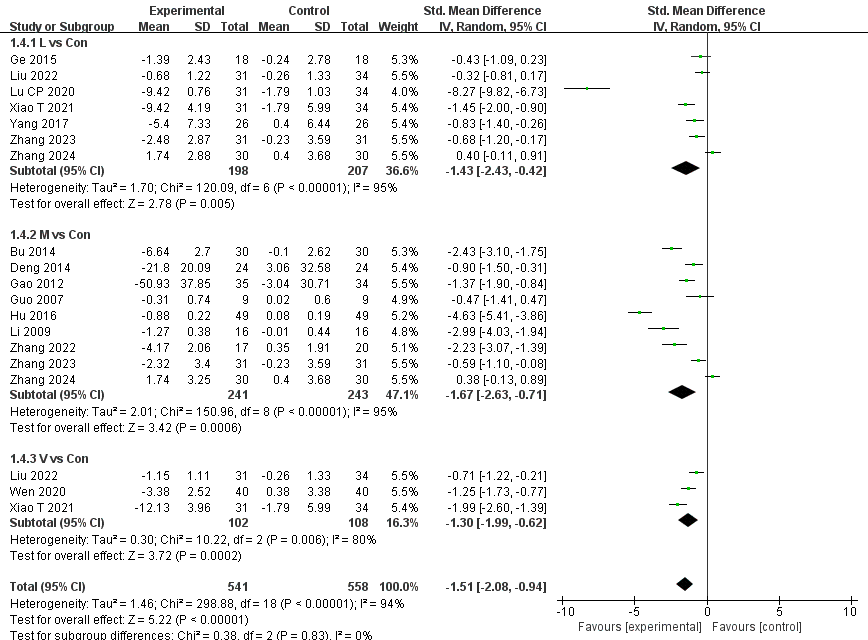


Appendix 2-4 forest plot of negative emotions

**Appendix 3. Subgroup analysis of internet addiction, depression, anxiety and negative emotions.**

| **Variable** | **Number of trials** | **Sample size** | | **Meta-Analysis** | | | | **Heterogeneity** | | |
| --- | --- | --- | --- | --- | --- | --- | --- | --- | --- | --- |
|  |  | **T** | **C** | **SMD** | **Lower** | **Upper** | **p^a^** | **I^2^(%)** | **Q** | **p^b^** |
| **Internet addiction** | 21 | 619 | 633 | -2.43 | -3.05 | -1.81 | - | 95 | 379.69 | <0.01 |
| **Population type** |  |  |  |  |  |  | 0.32 |  |  |  |
| *University* | 19 | 543 | 560 | -2.05 | -2.59 | -1.51 |  | 93 | 248.08 | <0.01 |
| *Middle school* | 2 | 76 | 73 | -6.19 | -14.30 | 1.91 |  | 99 | 92.97 | <0.01 |
| **Sample size** |  |  |  |  |  |  | 0.60 |  |  |  |
| *≤50* | 5 | 93 | 96 | -2.77 | -4.16 | -1.37 |  | 92 | 49.62 | <0.01 |
| *51-100* | 16 | 526 | 537 | -2.35 | -3.06 | -1.63 |  | 95 | 328.62 | <0.01 |
| **Intervention** |  |  |  |  |  |  | 0.64 |  |  |  |
| *Moderate-intensity exercise* | 9 | 274 | 276 | -2.80 | -3.89 | -1.72 |  | 96 | 181.67 | <0.01 |
| *High-intensity exercise* | 4 | 129 | 132 | -2.22 | -3.36 | -1.06 |  | 92 | 39.18 | <0.01 |
| *Light-intensity exercise* | 8 | 216 | 225 | -2.13 | -3.11 | -1.15 |  | 95 | 127.42 | <0.01 |
| **Intervention duration** |  |  |  |  |  |  | 0.05 |  |  |  |
| *≦8 weeks* | 7 | 195 | 198 | -1.66 | -2.54 | -0.78 |  | 93 | 83.68 | <0.01 |
| *> 8 weeks* | 14 | 424 | 435 | -2.86 | -3.70 | -2.03 |  | 95 | 284.07 | <0.01 |
| **Risk of bias** |  |  |  |  |  |  | 0.45 |  |  | <0.01 |
| *High* | 1 | 27 | 24 | -2.09 | -2.78 | -1.39 |  | - | - | - |
| *Low* | 20 | 592 | 609 | -2.45 | -3.10 | -1.81 |  | 95 | 377.17 | <0.01 |
| **Frequency** |  |  |  |  |  |  | 0.84 |  |  |  |
| *<3 times/week* | 4 | 110 | 122 | -2.59 | -4.26 | -0.92 |  | 96 | 69.98 | <0.01 |
| *≥3 times/week* | 17 | 509 | 511 | -2.40 | -3.10 | -1.71 |  | 95 | 309.60 | <0.01 |
| **Exercise Duration** |  |  |  |  |  |  | 0.002 |  |  |  |
| *≦60 minutes* | 13 | 376 | 382 | -1.54 | -2.02 | -1.05 |  | 88 | 102.65 | <0.01 |
| *>60 minutes* | 8 | 243 | 251 | -4.21 | -5.87 | -2.56 |  | 97 | 250.27 | <0.01 |
| **Depression** | 9 | 237 | 240 | -1.24 | -2.06 | -0.42 | - | 94 | 126.14 | <0.01 |
| **Population type** |  |  |  |  |  |  | 0.29 |  |  |  |
| *University* | 7 | 179 | 182 | -0.70 | -1.08 | -0.31 |  | 68 | 18.69 | <0.01 |
| *Middle school* | 2 | 58 | 58 | -2.98 | -7.24 | 1.27 |  | 98 | 44.11 | <0.01 |
| **Sample size** |  |  |  |  |  |  | 0.94 |  |  |  |
| *≤50* | 4 | 66 | 69 | -1.20 | -1.76 | -0.65 |  | 51 | 6.16 | 0.10 |
| *51-100* | 5 | 171 | 171 | -1.26 | -2.59 | 0.06 |  | 97 | 116.64 | <0.01 |
| **Intervention** |  |  |  |  |  |  | 0.05 |  |  |  |
| *Moderate-intensity exercise* | 7 | 176 | 179 | -1.52 | -2.62 | -0.42 |  | 95 | 113.90 | <0.01 |
| *Light-intensity exercise* | 2 | 61 | 61 | -0.34 | -0.70 | 0.02 |  | 0 | 0.04 | 0.85 |
| **Intervention duration** |  |  |  |  |  |  | 0.27 |  |  |  |
| *≦8 weeks* | 7 | 164 | 167 | -0.67 | -1.07 | -0.28 |  | 66 | 17.67 | <0.01 |
| *> 8 weeks* | 2 | 73 | 73 | -3.02 | -7.17 | 1.14 |  | 98 | 65.46 | <0.01 |
| **Frequency** |  |  |  |  |  |  | 0.55 |  |  |  |
| *<3 times/week* | 2 | 26 | 29 | -0.98 | -1.54 | -0.41 |  | 0 | 0.18 | 0.67 |
| *≥3 times/week* | 7 | 211 | 211 | -1.33 | -2.34 | -032 |  | 95 | 125.79 | <0.01 |
| **Exercise Duration** |  |  |  |  |  |  | <0.01 |  |  |  |
| *≦60 minutes* | 8 | 188 | 191 | -0.70 | -1.05 | -0.34 |  | 63 | 18.88 | <0.01 |
| *>60 minutes* | 1 | 49 | 49 | -5.15 | -5.98 | -0.42 |  | - | - |  |
| **Anxiety** | 12 | 330 | 342 | -1.94 | -2.81 | -1.08 | - | 95 | 239.85 | <0.01 |
| **Population type** |  |  |  |  |  |  | 0.40 |  |  |  |
| *University* | 10 | 272 | 284 | -1.39 | -2.04 | -0.74 |  | 91 | 104.25 | <0.01 |
| *Middle school* | 2 | 58 | 58 | -4.98 | -13.28 | 3.32 |  | 99 | 97.49 | <0.01 |
| **Sample size** |  |  |  |  |  |  | 0.53 |  |  |  |
| *≤50* | 4 | 66 | 69 | -1.65 | -2.45 | -0.84 |  | 73 | 11.31 | 0.01 |
| *51-100* | 8 | 264 | 273 | -2.10 | -3.28 | -0.92 |  | 97 | 223.11 | 0.01 |
| **Intervention** |  |  |  |  |  |  | 0.71 |  |  |  |
| *Moderate-intensity exercise* | 7 | 176 | 179 | -2.28 | -3.73 | -0.84 |  | 96 | 167.65 | <0.01 |
| *High-intensity exercise* | 1 | 31 | 34 | -1.89 | -2.48 | -1.30 |  | - | - | - |
| *Light-intensity exercise* | 4 | 123 | 129 | -1.46 | -2.78 | -0.14 |  | 95 | 63.10 | <0.01 |
| **Intervention duration** |  |  |  |  |  |  | 0.02 |  |  |  |
| *≦8 weeks* | 7 | 164 | 167 | -0.89 | -1.43 | -0.34 |  | 81 | 31.34 | <0.01 |
| *> 8 weeks* | 5 | 166 | 175 | -3.44 | -5.43 | -1.44 |  | 97 | 159.86 | <0.01 |
| **Frequency** |  |  |  |  |  |  | 0.70 |  |  |  |
| *<3 times/week* | 3 | 57 | 63 | -2.28 | -4.35 | -0.21 |  | 94 | 33.27 | <0.01 |
| *≥3 times/week* | 9 | 273 | 279 | -1.83 | -2.79 | -086 |  | 96 | 185.47 | <0.01 |
| **Exercise Duration** |  |  |  |  |  |  | 0.03 |  |  |  |
| *≦60 minutes* | 8 | 188 | 191 | -0.94 | -1.43 | -0.44 |  | 80 | 35.04 | <0.01 |
| *>60 minutes* | 4 | 142 | 151 | -4.01 | -6.67 | -1.36 |  | 98 | 153.80 | <0.01 |
| **Negative moods** | 19 | 541 | 558 | -1.51 | -2.08 | -0.94 |  | 94 | 298.88 | <0.01 |
| **Population type** |  |  |  |  |  |  | 0.57 |  |  |  |
| *University* | 17 | 483 | 500 | -1.36 | -1.88 | -0.84 |  | 92 | 212.10 | <0.01 |
| *Middle school* | 2 | 58 | 58 | -2.56 | -6.64 | 1.52 |  | 98 | 45.12 | <0.01 |
| **Sample size** |  |  |  |  |  |  | 0.72 |  |  |  |
| *≤50* | 5 | 84 | 87 | -1.36 | -2.25 | -0.47 |  | 84 | 25.48 | <0.01 |
| *51-100* | 14 | 457 | 471 | -1.57 | -2.26 | -0.87 |  | 95 | 272.85 | <0.01 |
| **Intervention** |  |  |  |  |  |  | 0.83 |  |  |  |
| *Moderate-intensity exercise* | 9 | 241 | 243 | -1.67 | -2.63 | -0.71 |  | 95 | 150.96 | <0.01 |
| *High-intensity exercise* | 3 | 102 | 108 | -1.30 | -1.99 | -0.62 |  | 80 | 10.22 | <0.01 |
| *Light-intensity exercise* | 7 | 198 | 207 | -1.43 | -2.43 | -0.42 |  | 95 | 120.09 | <0.01 |
| **Intervention duration** |  |  |  |  |  |  | 0.04 |  |  |  |
| *≦8 weeks* | 8 | 204 | 207 | -0.87 | -1.55 | -0.18 |  | 90 | 70.57 | <0.01 |
| *> 8 weeks* | 11 | 337 | 351 | -1.98 | -2.80 | -1.17 |  | 95 | 196.44 | <0.01 |
| **Frequency** |  |  |  |  |  |  | 0.29 |  |  |  |
| *<3 times/week* | 5 | 119 | 131 | -2.25 | -3.89 | -0.60 |  | 96 | 103.02 | <0.01 |
| *≥3 times/week* | 14 | 422 | 427 | -1.31 | -1.91 | -0.70 |  | 93 | 195.82 | <0.01 |
| **Exercise Duration** |  |  |  |  |  |  | <0.01 |  |  |  |
| *≦60 minutes* | 12 | 316 | 325 | -0.78 | -1.22 | -0.35 |  | 85 | 73.55 | <0.01 |
| *>60 minutes* | 7 | 225 | 233 | -2.81 | -4.04 | -1.57 |  | 96 | 140.45 | <0.01 |

T: experimental group; C: control group; p^a^ value for the between-group difference; p^b^ value for the heterogeneity within subgroups according to the Q test.

**Appendix 4.** Funnel plot of internet addiction, depression, anxiety and negative emotions in pairwise meta-analysis.


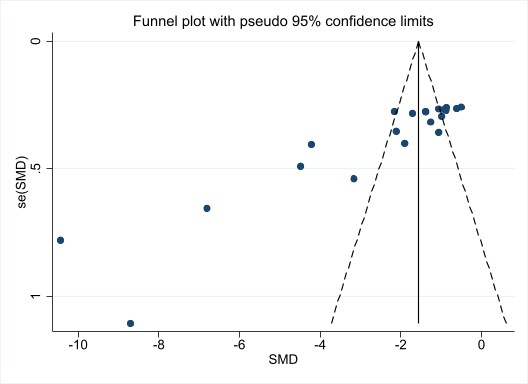


Appendix 4-1 funnel plot of internet addiction.


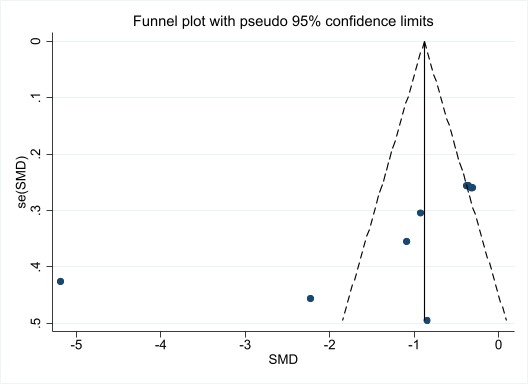


Appendix 4-2 funnel plot of depression.


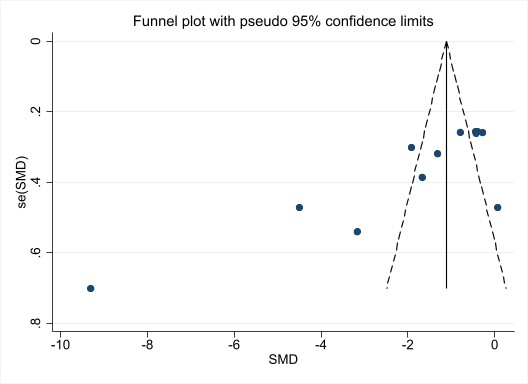


Appendix 4-3 funnel plot of anxiety.


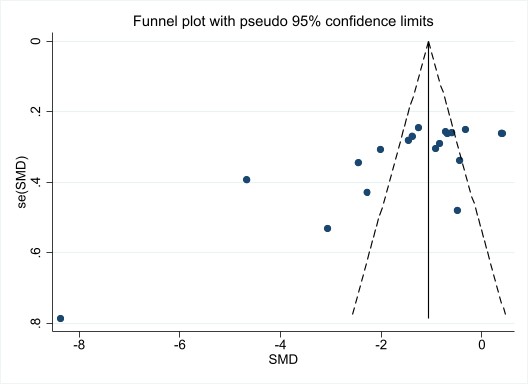


Appendix 4-4 funnel plot of negative emotions.

**Appendix 5.** Contributions of direct and indirect comparisons to NMA and the number of studies of each direct comparison of Internet addiction, depression, anxiety and negative emotions(A:Con;B:Light;C:Moderate;D:Vigorous;E:combined).


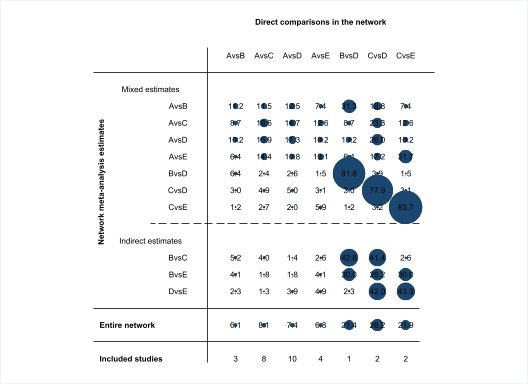


Appendix 5-1 Internet addiction


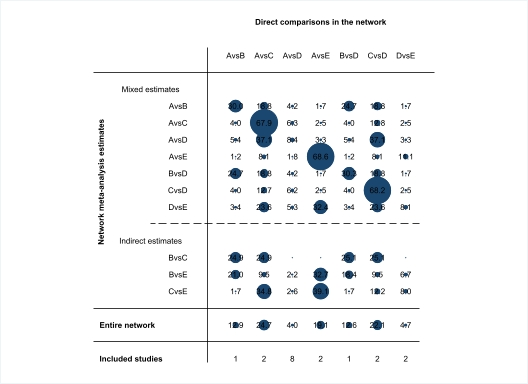


Appendix 5-2 Depression


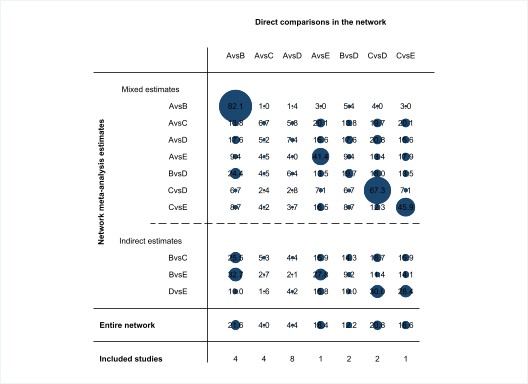


Appendix 5-3 Anxiety


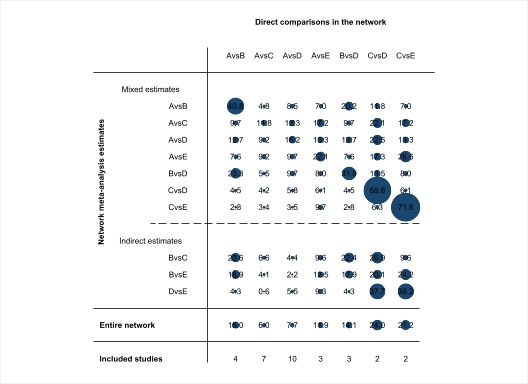


Appendix 5-4 Negative emotions

**Appendix 6.** Inconsistency of internet addiction, depression, anxiety and negative emotions tested by loop-specific heterogeneity estimates, inconsistency model and node splitting analysis.

| **loop-specific heterogeneity estimates** | | | | | | |
| --- | --- | --- | --- | --- | --- | --- |
| **Loop** | **IF** | **seIF** | **z_value** | **p_value** | **CI_95** | **Loop_Heterog_tau2** |
| **Internet addiction** | | | | | | |
| Con-Combined-M | 1.903 | 3.037 | 0.627 | **0.531** | (0.00,7.85) | 2.191 |
| Con-L-V | 0.76 | 2.388 | 0.318 | **0.75** | (0.00,5.44) | 1.868 |
| Con-L-M | 0.435 | 2.446 | 0.178 | **0.859** | (0.00,5.23) | 2.358 |
| **Depression** | | | | | | |
| Con-M-V | 1.727 | 1.613 | 1.071 | **0.284** | (0.00,4.89) | 1.57 |
| Con-L-M | 1.231 | 1.762 | 0.698 | **0.485** | (0.00,4.68) | 2.141 |
| Con-Combined-M | 0.404 | 2.804 | 0.144 | **0.885** | (0.00,5.90) | 2.373 |
| **Anxiety** | | | | | | |
| Con-Combined-M | 2.645 | 2.279 | 1.16 | **0.246** | (0.00,7.11) | 1.846 |
| Con-L-M | 0.937 | 2.641 | 0.355 | **0.723** | (0.00,6.11) | 2.995 |
| Con-L-V | 0.118 | 0.423 | 0.28 | **0.78** | (0.00,0.95) | 0 |
| **Negative emotions** | | | | | | |
| Con-Combined-M | 1.448 | 1.062 | 1.362 | **0.173** | (0.00,3.53) | 1.342 |
| Con-L-V | 0.715 | 2.45 | 0.292 | **0.771** | (0.00,5.52) | 1.405 |
| Con-L-M | 0.448 | 1.782 | 0.252 | **0.801** | (0.00,3.94) | 1.694 |

| **Inconsistency model** | | | | |
| --- | --- | --- | --- | --- |
|  | **Internet addiction** | **Depression** | **Anxiety** | **Negative emotions** |
| chi2 | 5.46 | 5.09 | 7.89 | 6.44 |
| Prob > chi2 | **0.4865** | **0.405** | **0.1627** | **0.4896** |

| **Node splitting analysis** | | | | | | | |
| --- | --- | --- | --- | --- | --- | --- | --- |
| **Side** | **Direct** | | **Indirect** | | **Difference** | |  |
|  | **Coef.** | **Std. Err.** | **Coef.** | **Std. Err.** | **Coef.** | **Std. Err.** | **P>\|z\|** |
| **Internet addiction** | | | | | | | |
| AB | -1.398235 | 1.447967 | -6.499999 | 4.631261 | 5.101764 | 4.852231 | **0.293** |
| AC | -2.391936 | 0.9086788 | -4.790747 | 2.8587 | 2.398811 | 2.999383 | **0.424** |
| AD | -2.595043 | 0.8124133 | -4.85974 | 3.129696 | 2.264697 | 3.233002 | **0.484** |
| AE | -2.20275 | 1.272358 | -5.513635 | 3.709004 | 3.310885 | 3.921197 | **0.398** |
| BD | 0.5177181 | 2.542131 | -1.683069 | 1.917783 | 2.200787 | 3.184345 | **0.489** |
| CD | -0.2821531 | 1.823188 | -0.0320984 | 1.384152 | -0.2500547 | 2.289091 | **0.913** |
| CE | -0.3476554 | 1.819637 | 0.5635071 | 2.009878 | -0.9111624 | 2.711088 | **0.737** |
| **Depression** | | | | | | | |
| AB | -0.4788475 | 1.609925 | -1.151592 | 3.020012 | 0.6727444 | 3.42644 | **0.844** |
| AC | -0.340126 | 1.057504 | -2.60212 | 2.186388 | 2.261994 | 2.428836 | **0.352** |
| AD | -1.249529 | 0.5544338 | -0.2669693 | 2.223116 | -0.9825599 | 2.291147 | **0.668** |
| AE | -0.6225765 | 1.051684 | -2.348315 | 1.495027 | 1.725739 | 1.827406 | **0.345** |
| BD | -0.4150011 | 1.609527 | -1.087746 | 3.020649 | 0.6727444 | 3.42644 | **0.844** |
| CD | 0.0076696 | 1.057346 | -2.254325 | 2.186618 | 2.261994 | 2.428836 | **0.352** |
| DE | -0.824871 | 1.015079 | 1.550098 | 1.398699 | -2.374969 | 1.728008 | **0.169** |
| **Anxiety** | | | | | | | |
| AB | -0.3651076 | 1.114109 | -6.097166 | 3.273069 | 5.732059 | 3.457376 | **0.097** |
| AC | -1.500391 | 1.212638 | -4.311518 | 3.546288 | 2.811127 | 3.747804 | **0.453** |
| AD | -1.87603 | 0.8821118 | -2.29054 | 3.280007 | 0.4145099 | 3.396451 | **0.903** |
| AE | -1.824496 | 2.455405 | -4.35631 | 5.003491 | 2.531814 | 5.572643 | **0.65** |
| BD | 0.5599096 | 1.633528 | -2.736183 | 1.794845 | 3.296093 | 2.426844 | **0.174** |
| CD | 0.0777192 | 1.747577 | -0.3360117 | 1.937878 | 0.4137309 | 2.609472 | **0.874** |
| CE | -1.00958 | 2.453407 | 1.522234 | 5.00643 | -2.531814 | 5.572643 | **0.65** |
| **Negative emotions** | | | | | | | |
| AB | -0.8012535 | 0.8540479 | -2.747223 | 1.564348 | 1.94597 | 1.782307 | **0.275** |
| AC | -1.525104 | 0.6667545 | -2.696448 | 2.092791 | 1.171345 | 2.196514 | **0.594** |
| AD | -1.517363 | 0.5602449 | -1.835743 | 1.65396 | 0.3183795 | 1.746195 | **0.855** |
| AE | -1.360057 | 1.00307 | -3.365401 | 2.606209 | 2.005344 | 2.792893 | **0.473** |
| BD | 0.4378259 | 0.9873635 | -1.492875 | 1.25562 | 1.930701 | 1.597119 | **0.227** |
| CD | 0.0240789 | 1.240965 | 0.1229651 | 0.9843329 | -0.0988862 | 1.58395 | **0.95** |
| CE | -0.4671922 | 1.229351 | 0.9187033 | 1.688434 | -1.385895 | 2.08809 | **0.507** |

**Appendix 7.** Network forest.


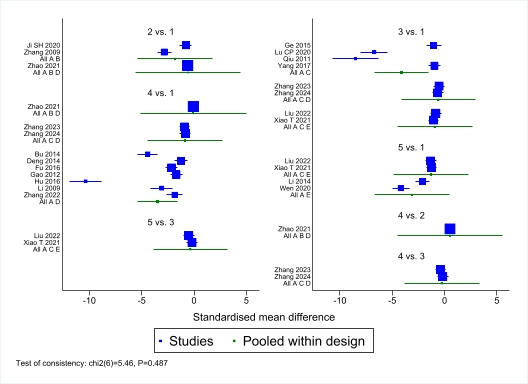


Appendix 6-1 internet addiction


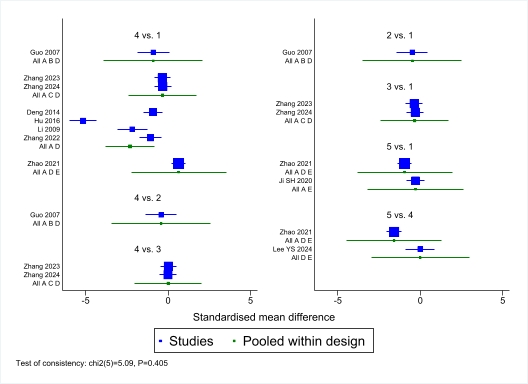


Appendix 6-2 Depression


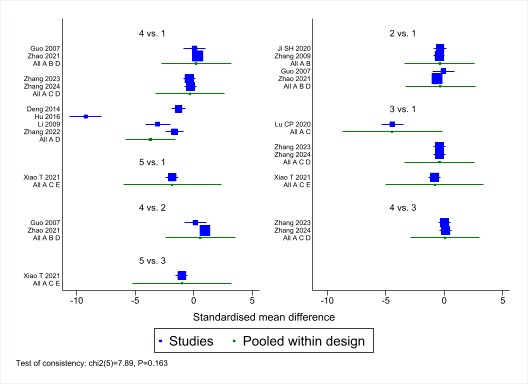


Appendix 6-3 Anxiety


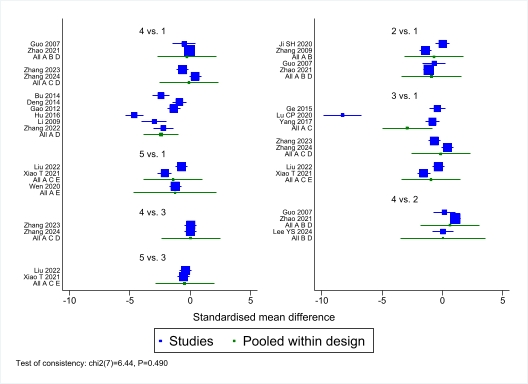


Appendix 6-4 Negative emotions

**Appendix 8.**Forest plots of eligible comparisons of Internet addiction, depression, anxiety and negative emotions.


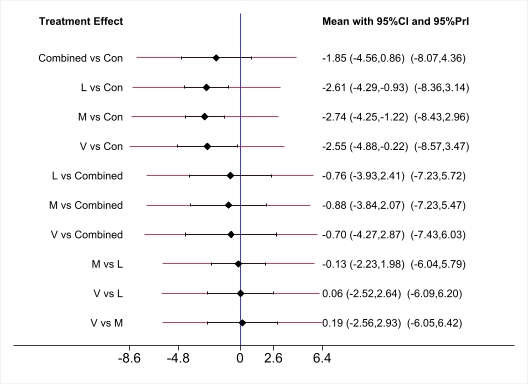


Appendix 8-1 internet addiction


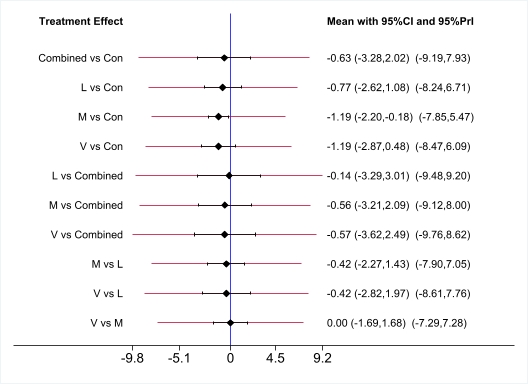


Appendix 8-2 Depression


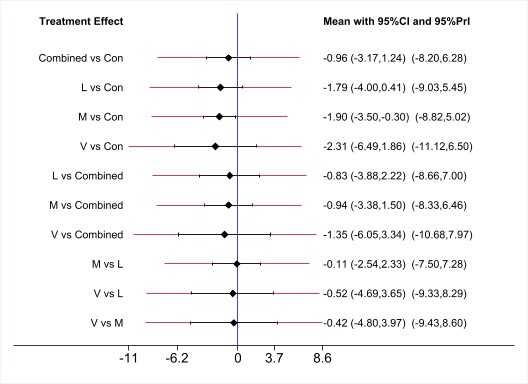


Appendix 8-3 Anxiety

**
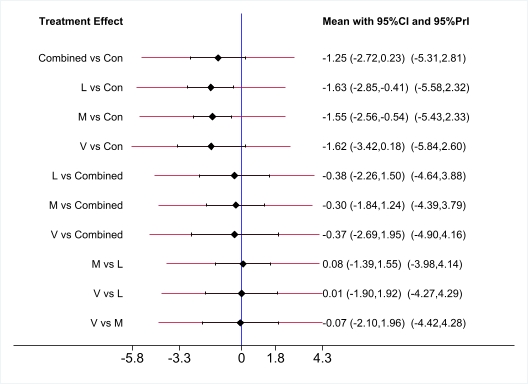
**

Appendix 8-4 Negative emotions

**Appendix 9.** Area under the curve for cumulative ranking probability of each intervention on internet addiction, depression, anxiety and negative emotions.


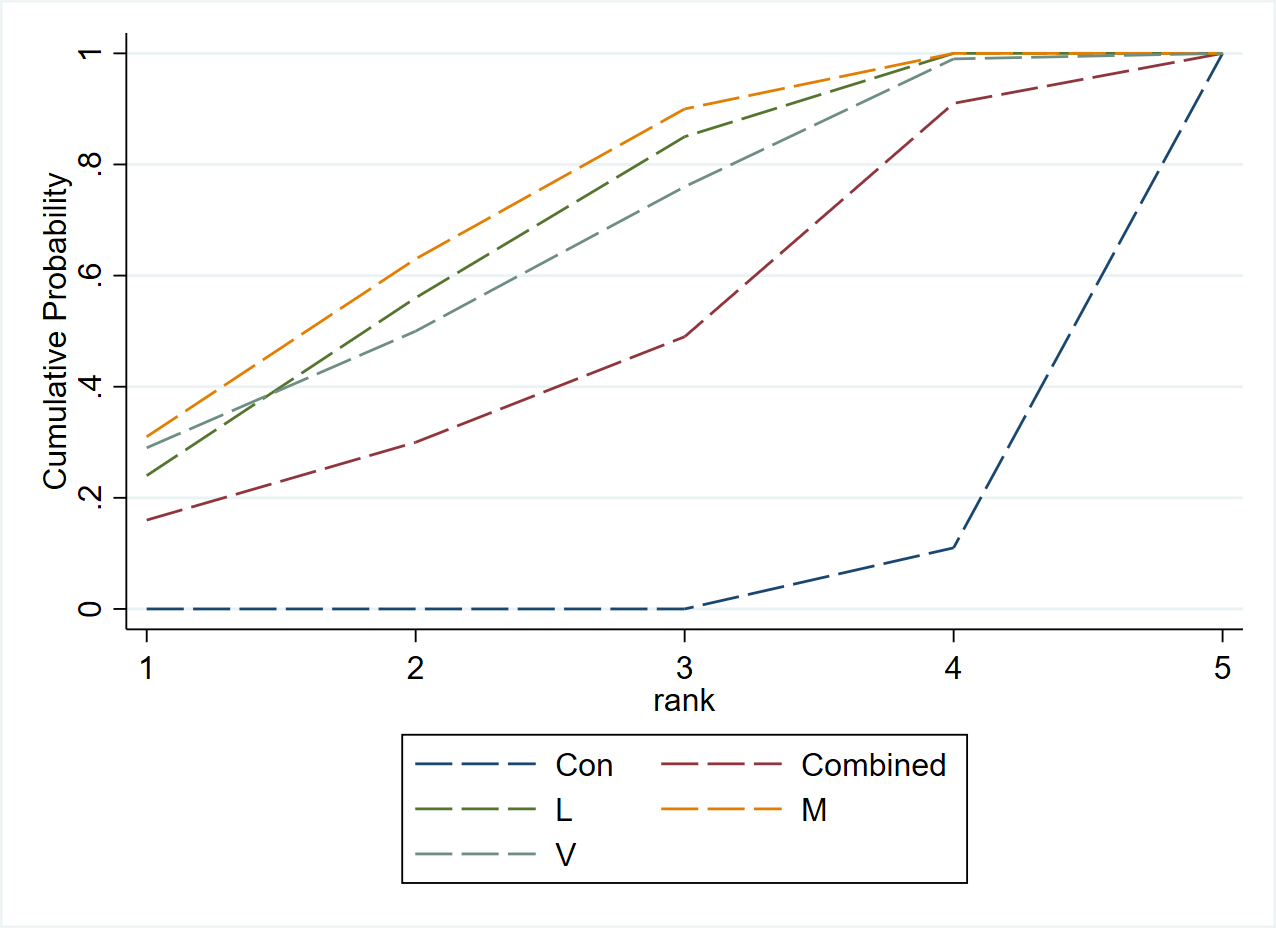


Appendix 9-1 internet addiction


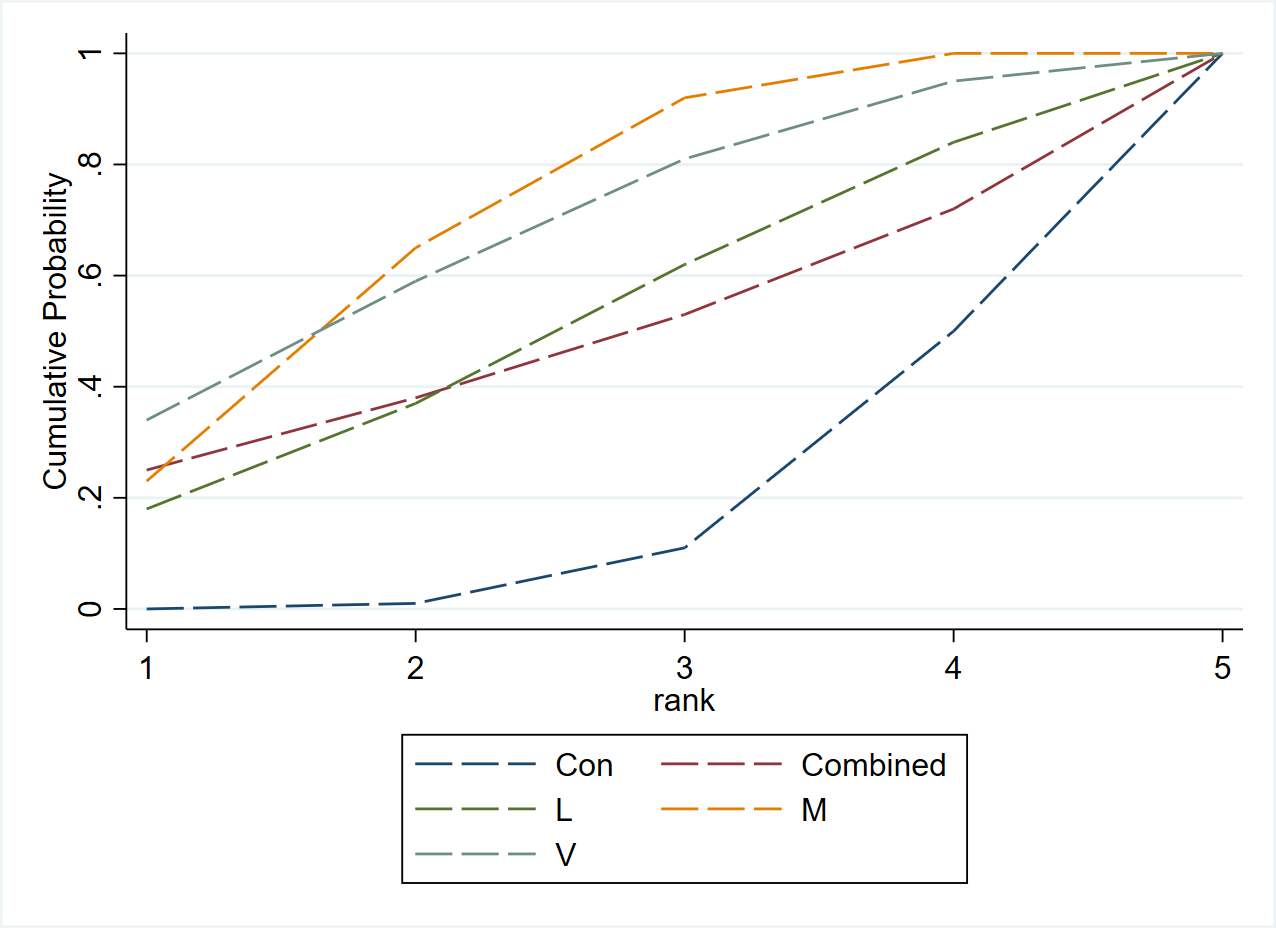


Appendix 9-2 depression


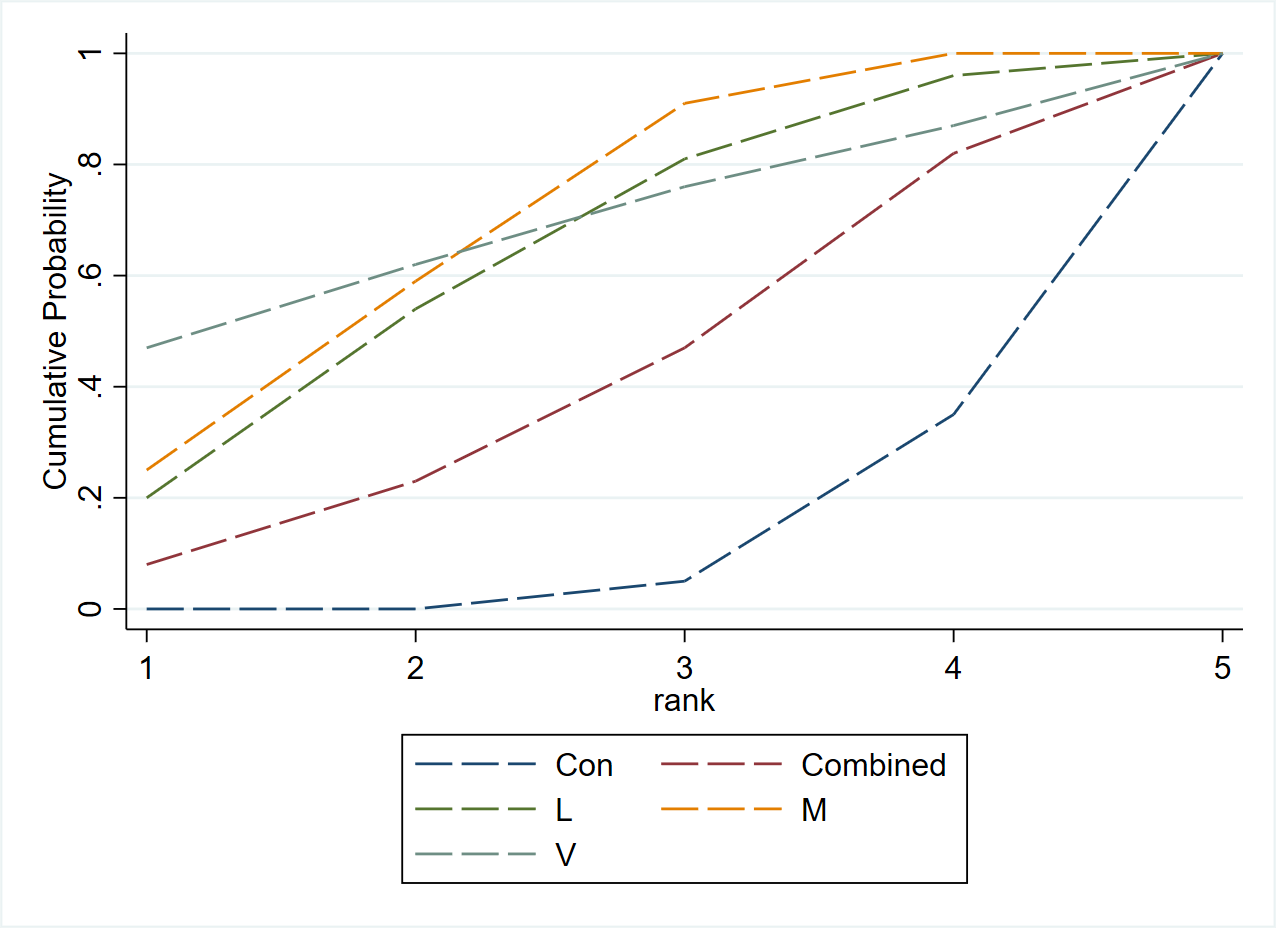


Appendix 9-3 Anxiety


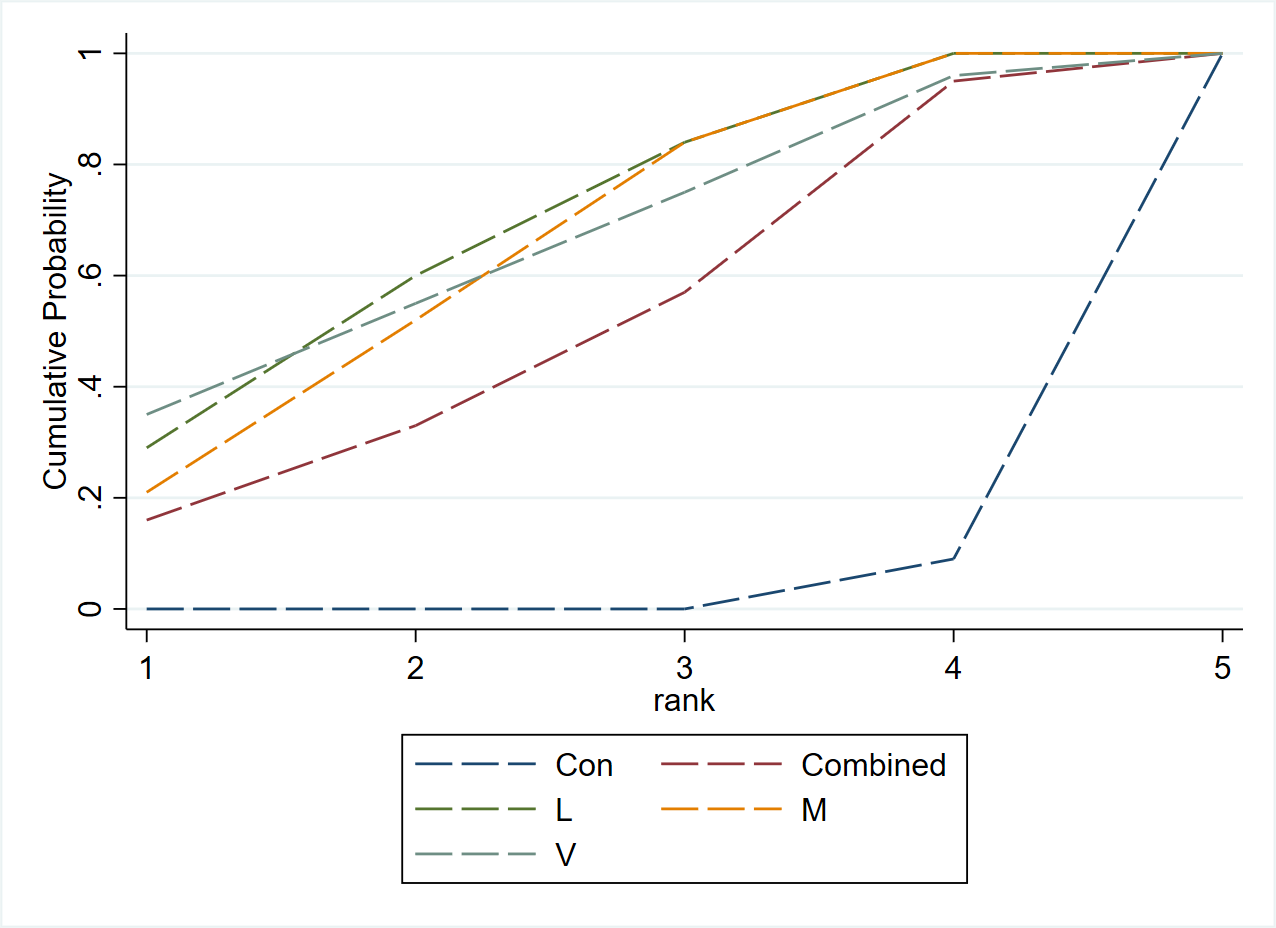


Appendix 9-4 Negative emotions

**Appendix 10.** The funnel plot graphics of internet addiction, depression, anxiety and negative emotions in NMA.


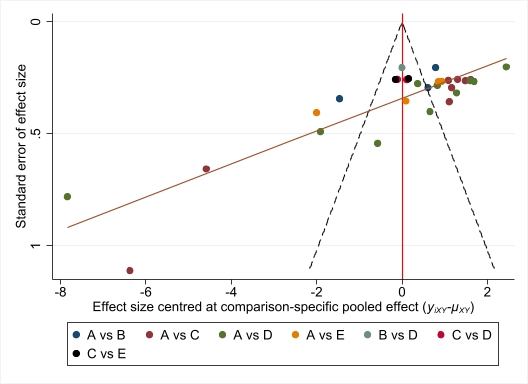


Appendix 10-1 Internet addiction


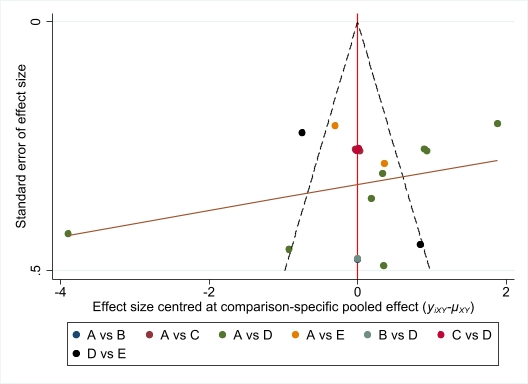


Appendix 10-2 Depression


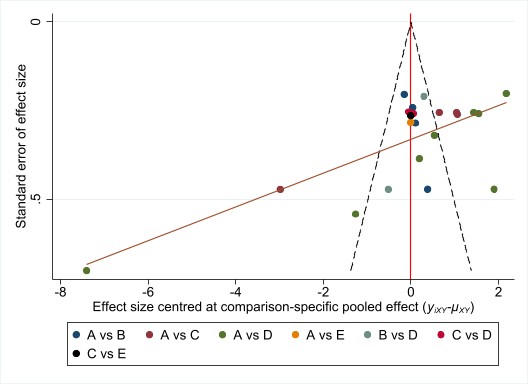


Appendix 10-3 Anxiety


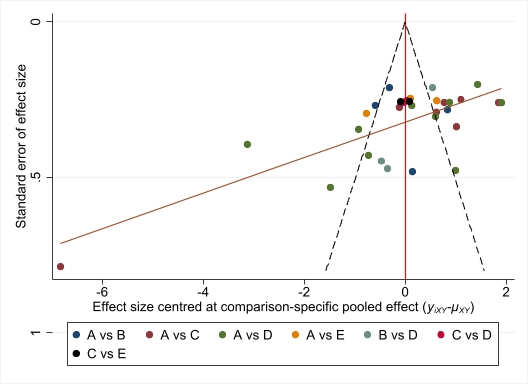


Appendix 10-4 Negative emotions

**Appendix 11.** GRADE assessment

**1. Summary of study limitations of the included studies.**

The colours of the line indicate the summative ROB assessment of each comparison based on ROB assessment of each included studies (low ROB comparison [green], moderate ROB comparison [yellow] and high ROB comparison [red]).

| 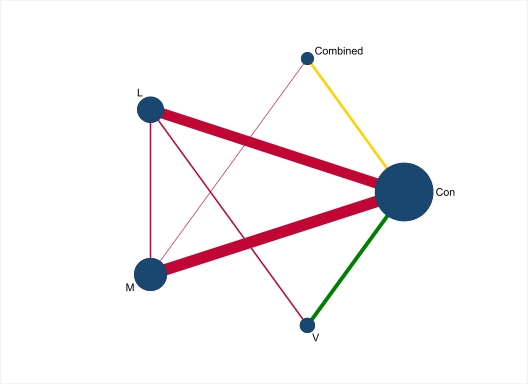 | 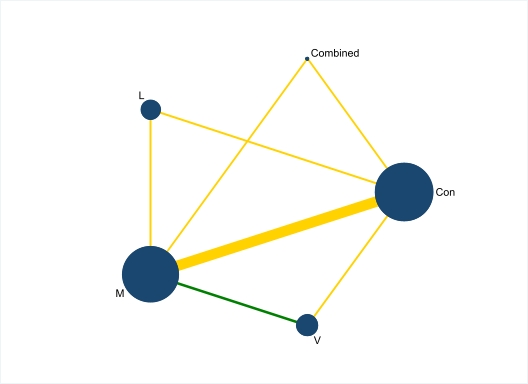 |
| --- | --- |
| Internet addiction | Depression |
| 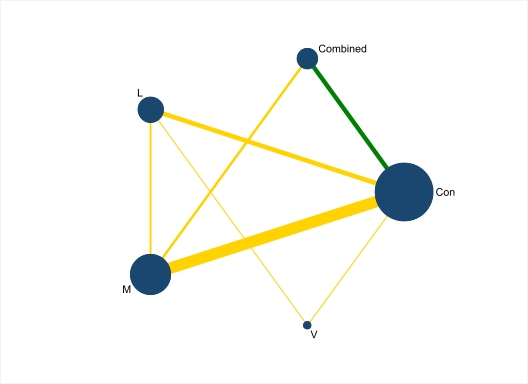 | 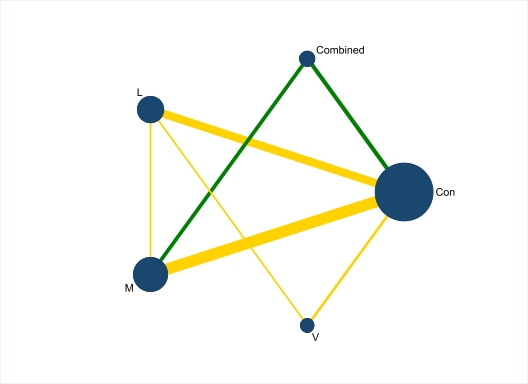 |
| Anxiety | Negative emotions |

Appendix 11-summary of tudy limitations

**2. Contribution of ROB comparisons to each network estimate.**

Based on the above assessment of ROB for each comparison and the contributions of direct and indirect comparisons to all network estimates, the following bar graphs show the percentage of low, moderate and high ROB contributions for each network estimate.


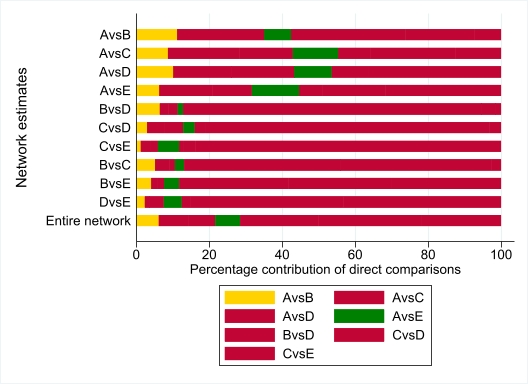


Appendix 11-2 Internet addiction


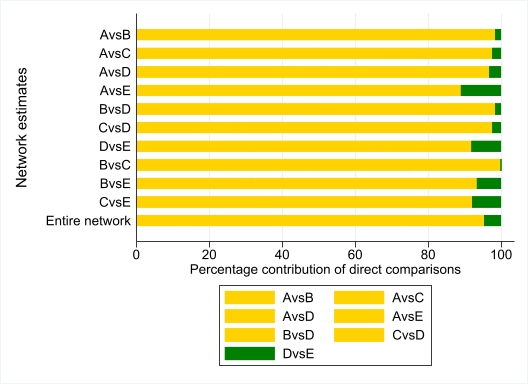


Appendix 11-3 Depression


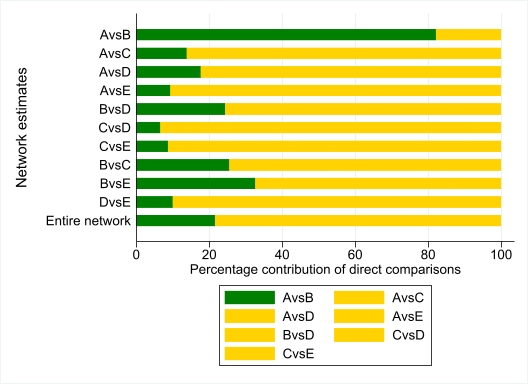


Appendix 11-4 Anxiety


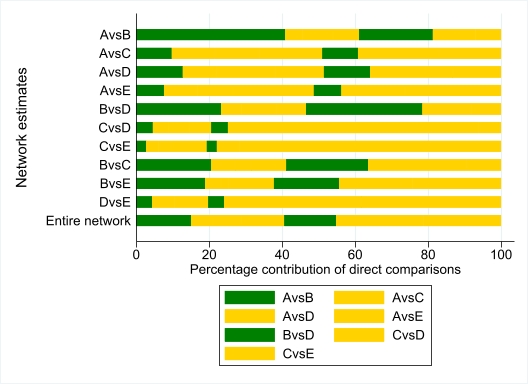


Appendix 11-5 Negative emotions

1. **Table of reasons for downgrading.**

Based on all the above information, we GRADE each network estimate according to the following criteria.

**(1) Study limitations**

Low risk (green)/unclear risk (yellow)/high risk (red) of each direct comparison was given a weight of 0/- 1/-2, respectively. And then the risk value of each comparison and SUCRA probability sorting result were calculated according to the contribution to ROB comparisons to each network estimate. It would be downgraded by one level when the risk value is between -1.5 and -0.6 (indicated by “Study limitations”) and two level when the risk value is less than -1.6 (indicated by “Study limitations^2^”).

**(2) Indirectness**

It was assessed by determining whether the study population, type of intervention, and results were directly relevant to the purpose of this meta-analysis.

**(3) Inconsistency**

In this project, we examined heterogeneity and inconsistency. Heterogeneity between each pair of direct comparisons was evaluated by whether the prediction interval of the pairwise forest plots (Appendix 8) crossed the invalid line, it was judged as highly heterogeneous if crossed (indicated by “Heterogeneity”). Inconsistency between direct and indirect comparisons was evaluated by loop-specific heterogeneity estimates and node splitting analysis (indicated by “Inconsistency”). The stability of the SUCRA probability sorting result is evaluated according to global inconsistency.

**(4) Imprecision**

Imprecision was evaluated by appropriate sample sizes, a sample size below 400 would be downgraded by one level. The total sample size in this study was over 400, so this project was not downgraded. The Imprecision of the ranking results is evaluated according to the gap between the ranking values. If the gap between the ranking values of each treatment is small, which indicating a poor stability, the intervention would be downgraded by one leve.

**(5) Publication bias**

Using Begg's of pairwise meta-analysis, the intensity of the intervention with publication bias was reduced by one level, and the group compared to this intensity was also reduced by one level

Although the NMA comparison-adjusted funnel plot did not suggest presence of overall publication bias, it was subjective evaluation and we cannot completely rule out the possibility that some studies are still missing. Therefore, for the group directly compared with CON, the Begg’s test of pairwise meta-analysis was used to evaluate whether there was publication bias. Considering that the small sample size of groups compared with non-CON may lead to publication bias, all groups compared with non-CON were downgraded by one level.

| **Internet addiction** | | | |
| --- | --- | --- | --- |
| **Comparison** | **Nature of the evidence** | **Confidence** | **Downgrading due to** |
| AB | Mixed | Low | Study limitations^2^, |
| AC | Mixed | Very low | Study limitations^2,^Publication bias |
| AD | Mixed | Very low | Study limitations^2^,Heterogeneity |
| AE | Mixed | Very low | Study limitations^2^,Heterogeneity |
| BD | Mixed | Very low | Study limitations^2^ |
| CD | Mixed | Low | Study limitations^2^ |
| CE | Mixed | Low | Study limitations^2^ |
| BC | Indirect | Low | Indirectness ,Publication bias |
| BE | Indirect | Low | Indirectness,Publication bias |
| DE | Indirect | Low | Indirectness,Publication bias |
|  |  |  |  |
| Ranking of treatments |  | Low | Study limitations^2^ |

| **Depression** | | | |
| --- | --- | --- | --- |
| **Comparison** | **Nature of the evidence** | **Confidence** | **Downgrading due to** |
| AB | Mixed | Low | Study limitations,Publication bias |
| AC | Mixed | Low | Study limitations,Publication bias |
| AD | Mixed | Low | Study limitations,Heterogeneity |
| AE | Mixed | Low | Study limitations,Publication bias |
| BD | Mixed | Moderate | Study limitations |
| CD | Mixed | Moderate | Study limitations |
| DE | Mixed | Low | Study limitations,Publication bias |
| BC | Indirect | Low | Indirectness ,Publication bias |
| BE | Indirect | Low | Indirectness,Publication bias |
| CE | Indirect | Low | Indirectness,Publication bias |
|  |  |  |  |
| Ranking of treatments |  | Moderate | Study limitations |

| **Negative emotions** | | | |
| --- | --- | --- | --- |
| **Comparison** | **Nature of the evidence** | **Confidence** | **Downgrading due to** |
| AB | Mixed | Moderate | Publication bias |
| AC | Mixed | Low | Study limitations,Heterogeneity |
| AD | Mixed | Low | Study limitations,Heterogeneity |
| AE | Mixed | Low | Study limitations,Publication bias |
| BD | Mixed | Moderate | Publication bias |
| CD | Mixed | Moderate | Study limitations |
| CE | Mixed | Moderate | Study limitations |
| BC | Indirect | Low | Indirectness ,Publication bias |
| BE | Indirect | Low | Indirectness,Publication bias |
| DE | Indirect | Low | Indirectness,Publication bias |
|  |  |  |  |
| Ranking of treatments |  | Moderate | Study limitations |

| **Anxiety** | | | |
| --- | --- | --- | --- |
| **Comparison** | **Nature of the evidence** | **Confidence** | **Downgrading due to** |
| AB | Mixed | Moderate | Publication bias |
| AC | Mixed | Low | Study limitations,Publication bias |
| AD | Mixed | Low | Study limitations,Heterogeneity |
| AE | Mixed | Moderate | Study limitations |
| BD | Mixed | Low | Study limitations,Publication bias |
| CD | Mixed | Moderate | Study limitations |
| CE | Mixed | Moderate | Study limitations |
| BC | Indirect | Low | Indirectness ,Publication bias |
| BE | Indirect | Low | Indirectness,Publication bias |
| DE | Indirect | Low | Indirectness,Publication bias |
|  |  |  |  |
| Ranking of treatments |  | Moderate | Study limitations |

**Reference**

Salanti G, Del Giovane C, Chaimani A, et al. Evaluating the quality of evidence from a network meta-analysis[J]. PLoS One, 2014, 9(7): e99682.
